# A Cluster Randomized Clinical Trial to Improve Prescribing Patterns in Ambulatory Pediatrics

**DOI:** 10.1371/journal.pctr.0020025

**Published:** 2007-05-18

**Authors:** Robert L Davis, Jeffrey Wright, Francie Chalmers, Linda Levenson, Julie C Brown, Paula Lozano, Dimitri A Christakis

**Affiliations:** 1 Department of Pediatrics, University of Washington School of Medicine, Seattle, Washington, United States of America; 2 Department of Epidemiology, University of Washington School of Public Health, Seattle, Washington, United States of America; 3 Child Health Institute, University of Washington School of Medicine, Seattle, Washington, United States of America; 4 Skagit Pediatrics Medical Group, Skagit Valley, Washington, United States of America

## Abstract

**Objectives:**

Having shown previously that an electronic prescription writer and decision support system improved pediatric prescribing behavior for otitis media in an academic clinic setting, we assessed whether point-of-care delivery of evidence could demonstrate similar effects for a wide range of other common pediatric conditions.

**Design:**

Cluster randomized controlled trial.

**Setting:**

A teaching clinic/clinical practice site and a primary care pediatric clinic serving a rural and semi-urban patient mix.

**Participants:**

A total of 36 providers at the teaching clinic/practice site and eight providers at the private primary pediatric clinic.

**Intervention:**

An evidence-based message system that presented real-time evidence to providers based on prescribing practices for acute otitis media, allergic rhinitis, sinusitis, constipation, pharyngitis, croup, urticaria, and bronchiolitis.

**Outcome measures:**

The proportion of prescriptions dispensed in accordance with evidence.

**Results:**

The proportion of prescriptions dispensed in accordance with evidence improved four percentage points, from 38% at baseline to 42% following the intervention. The control group improved by one percentage point, from 39% at baseline to 40% at trial's conclusion. The adjusted difference between the intervention and control groups was 8% (95% confidence interval 1%, 15%). Intervention effectiveness did not decrease with time.

**Conclusion:**

For common pediatric outpatient conditions, a point-of-care evidence-based prescription writer and decision support system was associated with significant improvements in prescribing practices.

## Editorial Commentary

**Background:** Computerized systems for managing health-care information, such as medical records and prescriptions, have the potential to improve medical care. These improvements could come about as a result of embedding software within a medical record system that alerts clinicians to evidence that is relevant to the care they are providing. For example, such a system might deliver a pop-up reminder that informs a clinician about a potential prescribing error, or that the prescription ordered is not supported by recent evidence. Systematic reviews of randomized controlled trials evaluating the benefits of such systems have shown that computerized feedback and reminder systems can improve clinician behavior. However, much of this evidence comes from academic clinics caring for adults, and there is not very much evidence available on children or from community-based, nonacademic clinical practices. The researchers here wanted to evaluate whether a computerized system providing clinical decision support at the time of electronic prescribing could improve prescribing in pediatric primary care. In order to test this, the researchers carried out a cluster randomized trial. This means that individual health-care providers were randomized to receive evidence-based prompts via the computerized system or not, depending on which arm of the trial they were randomized to, but outcome data for the trial were collected at the level of the individual patient's prescription. Pop-up prompts were provided for eight medical conditions common in pediatric primary care, and alerted the provider to a summary of the evidence that supported or refuted the prescription that the provider was about to make. The primary outcome in the trial was the change in proportion of prescriptions dispensed in accordance with evidence, over the course of the trial.

**What the trial shows:** In the trial, 36 pediatric health-care providers were randomized at one site, where the trial was carried out over 50 months, and eight at another, where the trial lasted for 18 months. At the start of the trial, 38% of prescriptions in the intervention group were in accordance with the evidence, and 39% of prescriptions in the control group. At the end of the trial, 42% of prescriptions in the intervention group were in accordance with evidence, as compared to 40% of prescriptions in the control group. The difference in prescribing behavior change over the course of the trial between intervention and control groups was statistically significant, once adjusted for the clustering of data by the individual providers.

**Strengths and limitations:** In this trial, health-care providers were randomized, rather than patients. This method (cluster randomization) is probably the most appropriate method to carry out a trial such as this, because it reduces the chance of contamination (i.e., that patients not assigned to the intervention might receive some of its benefits). One limitation is the small number of providers that were recruited into the trial; another is that many of these in fact practiced or had recently practiced in academic medicine rather than community-based, nonacademic clinical practices. This limits the ability to generalize from these findings to a nonacademic setting. Finally, the trial was planned with the intention of evaluating the ability of computerized systems to achieve health-care provider behavior change. Therefore, data were analyzed by lumping together outcomes for many different medical conditions. This means that the computerized prompt system may not necessarily have been that successful in achieving improvements in prescribing for any individual condition.

**Contribution to the evidence:** Systematic reviews of the effectiveness of computerized decision support systems of this kind have found some evidence that such systems can improve the behavior of health-care providers. This study adds data showing that the reminder system studied here resulted in moderate improvements in prescribing within a pediatric primary care setting.

## INTRODUCTION

Health information technologies offer substantial promise to improve health care [1]. Electronic medical records (EMRs), decision support systems (DSSs), and computerized provider order entry (CPOE) offer the potential to reduce practice variation, improve access to patient data, increase efficiency of documentation, provide decision support for practitioners, and deliver educational materials to patients [2]. In studies involving hospitalized adults, CPOE systems have reduced errors; increased accuracy, readability, and completeness; facilitated decision support; and reduced costs [3–18].

In one outpatient setting with a comprehensive EMR system, provider management decisions and choice of medications were influenced by alerts for routine screening, for abnormal physical examination parameters, and for potential medication side effects [19–22]. Many other studies that have evaluated the impact of EMR systems in the outpatient setting have focused on the use of computer-generated provider or patient reminders to improve preventive care services, and have demonstrated a varying range of results [23–31]. However, systematic reviews of studies that have assessed computerized DSSs providing reminders and feedback to health-care providers (HCPs) have shown them to make relatively modest improvement in prescribing practices [32,33].

In pediatrics, CPOE with DSS has reduced errors in the treatment of seriously ill, hospitalized children. In Tennessee, medication prescribing errors in a critical care unit fell from a rate of 30 per 100 orders to 0.2 per 100 orders after implementation of CPOE [14]. In Salt Lake City, Utah, implementation of an anti-infective decision support tool in a pediatric intensive care unit reduced the rate of drug prescribing errors requiring pharmacist intervention by more than half [34]. Computer-based documentation has also been shown to improve the delivery of pediatric preventive services [2,35]. Nevertheless, even though 61.2 million visits each year are made by children to physicians in the office-based setting, and 26% of these visits result in an antibiotic prescription [36], adoption and dissemination of CPOE with DSS has been slow. There are many reasons for this, but at least some of the reasons are that the cost of these systems can be substantial, while the feasibility and effectiveness of CPOE and/or decision support in the ambulatory pediatric setting are largely unknown.

We previously demonstrated the ability of an electronic prescription writer and DSS to improve pediatric prescribing behavior for otitis media in an academic clinic setting [37]. Using a “homegrown” DSS we were able to demonstrate a 34% greater reduction in prescribing for otitis media among providers given evidence-based messages at the time of prescription writing compared to providers not given such messages. These findings led us to address the question of whether such a system could demonstrate a similar effect for a wide range of other common conditions typically seen by pediatricians, and equally important, whether such a system could work in a community-based, nonacademic practice setting. In this paper we present the results of a cluster randomized clinical trial designed to answer these questions.

## METHODS

### Design

This was a cluster randomized clinical trial of provider behavior change. We measured prescribing behavior in both the intervention and control groups before and after the introduction in the intervention group of a DSS providing evidence at the time of electronic prescribing. This approach enabled us to measure the independent effects of the intervention, while controlling for baseline differences in prescribing behavior and other temporal trends unrelated to our intervention.

In this study the unit of intervention was the provider. We chose this design because a cluster randomized clinical trial is the strongest study design available to assess the effect of a DSS upon provider behavior, as it directly compares providers receiving the intervention to those not receiving the intervention. Direct contamination between providers was likely to be minimal because, as detailed below, the DSS provided privately viewed messages that only briefly engaged the practitioners and their patients. Had we randomized patients instead, it would have resulted in providers taking care of both patients receiving the intervention and others not receiving the intervention, with the strong possibility of diluting the intervention effect.

### Setting

This study was conducted at two clinical sites. One was the Pediatric Care Center at the University of Washington (PCC), an outpatient teaching clinic for pediatric residents and a clinical practice site staffed by full-time pediatric providers. The other site was Skagit Pediatrics (SP), a primary care pediatric clinic serving a rural and semi-urban patient mix approximately 60 mi north of the Seattle metropolitan area.

At the start of the study period at PCC, care was provided by 29 resident physicians, two nurse practitioners, and seven attending physicians, each with their own patient panels. At SP, there were eight physicians and two nurse practitioners, also each with their own patient panels. Both clinics adopted a computerized patient flow manager developed by one of us (JAW); this system was described in detail in our earlier publication [35]. At PCC, a computer workstation was placed in physician work areas and nursing stations and connected to a server via a local area network. At SP, because of limited space availability in exam rooms, providers were equipped with wireless handheld computers (either personal digital assistants or pen-based tablet computers) connected via the local area network to a server.

An electronic prescription writer was developed to interface with the computerized patient flow manager. To prescribe a medication, a provider first selected the patient's name and then the patient's medication, indication, dosage, and, finally, the duration. The patient's weight was entered by the nurse during check-in. A paper copy of the prescription was printed for the patient and was also attached to each medical record.

Providers were trained on the network, and then a 6-mo period prior to randomization was used at each site to collect baseline prescribing behavior for all providers. When the intervention started, paper prescriptions were either removed (at PCC) or actively discouraged (at SP).

### Participants

**Figure 1:**
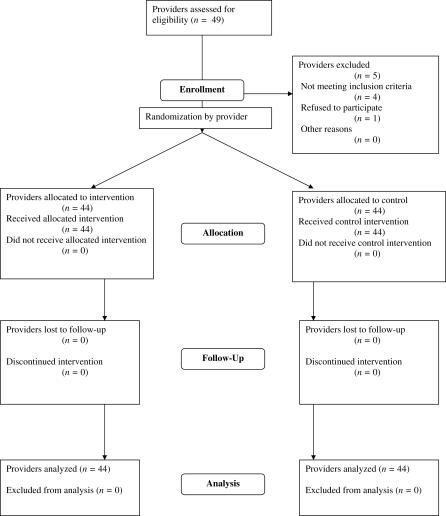
Recruitment, Randomization, and Analysis of Providers in Study

The participants in the study were the 36 HCPs at PCC and the eight HCPs at SP (Figure 1). Study investigators at each site were excluded from participation. The protocol for both sites was approved by the University of Washington Institutional Review Board. Consent to participate in the study was given by the providers; individual consent from patients was not required.

### Randomization

At each site, the unit of randomization was the HCP. We used a stratified randomization process to randomly assign providers to either the intervention arm or the control arm. Specifically, for each condition (otitis media, croup, etc.), providers were first stratified by the number of prescriptions they wrote in the baseline period, in order to roughly equalize the number of patients seen by providers and prescriptions written in the intervention and control arms. Then, within strata of high or low number of prescriptions written, HCPs were randomly assigned to receive evidence-based medicine prompts or not. In both clinics, providers could have been randomly assigned to receive anywhere from none to all of the evidence-based prompts; after randomization it turned out that all providers received at least one evidence-based prompt.

Random numbers for allocation were generated by computer, and were concealed until interventions were assigned. This process was overseen by the research coordinator (L. L.) in conjunction with the investigator in charge of the data structure (J. W.). L. L. enrolled participants and assigned participants to their groups based on the randomization. Participants were not informed in advance of the study as to which conditions were being investigated, and hence were blinded to the intervention. However, based on the nature of the intervention, HCPs were (theoretically) able to determine to which evidence screens they were randomized by discussions with other HCPs.

In 2000 and 2001, additional residents were randomized as they joined PCC as interns. After baseline data collection for these residents, they were randomized as the others to either the intervention or control groups, and then followed in the same manner as the other providers until study conclusion.

### Interventions and Conditions Studied

For each condition studied, providers in the treatment arm were shown pop-up “alert” screens, based on their selection of medication, indication, or duration. The first screen contained a short summary of the evidence either supporting or refuting the current choice of medication, indication, or duration. The provider could then choose to (i) view more information about this evidence, (ii) view the abstract of the article from which the evidence was derived, (iii) view a scanned PDF version of the article, or (iv) have the reference E-mailed to them for later viewing. Table 1 shows examples of the first screens shown to providers for otitis media and allergic rhinitis (a screenshot of how this message appeared is shown in Figure S1). In the vast majority of cases, providers did not venture past the first screen during the process of writing a prescription.

**Table 1:**
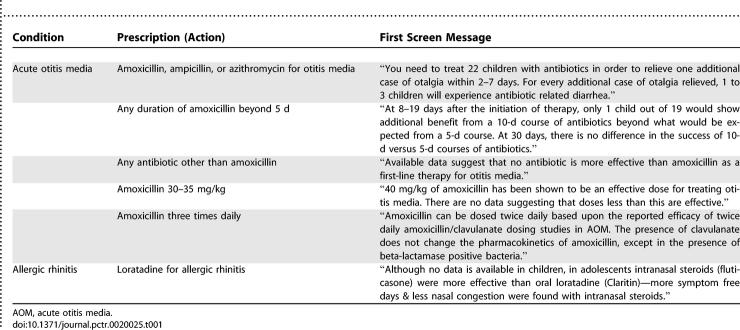
Example Summaries of Actions Triggering First-Level Evidence Screen for Two Selected Conditions

The conditions included in the intervention were acute otitis media, allergic rhinitis, sinusitis, constipation, pharyngitis, croup, urticaria, and bronchiolitis. For the outcome of otitis media, a small percentage of the data includes data previously published in the earlier report by Christakis et al. Excluding these data did not affect our results or conclusions, and we have elected to include these data so that we are able to address the consistency of effect among the two intervention sites. The full details of the information provided in the evidence screens are available from the authors.

### Outcomes

#### Main analysis.

Our primary outcome was changed physician behavior in accordance with the intervention message screens. Our primary measure assessed all of the interventions considered together, in order to answer the question, “Can we influence provider prescribing behavior by providing ‘just-in-time,' evidence-based prompts?” We also looked at the effect of the message screens upon the separate outcomes of (i) otitis media, (ii) allergic rhinitis, and (iii) a combined category of the other (less common) conditions. These categories were chosen a priori, as these conditions were the ones found to be most frequently evaluated in the clinic during the study planning stages (and were not necessarily the conditions for which medications were most frequently later prescribed). All analyses were performed by “intention to treat.”

Bronchiolitis was studied separately. This was necessary because the “baseline” period (used for randomization and to measure change from baseline) did not include the most recent past bronchiolitis season, and hence change from baseline could not be measured in the analysis. Instead, for bronchiolitis we compared the behavior of providers receiving the intervention directly to the behavior of control providers.

#### Subanalyses.

We performed two subanalyses. In our first subanalysis we added a “one click” option for allergic rhinitis and otitis media that allowed a provider to rapidly accept a pre-written electronic prescription corresponding to the “correct” message presented on the screen. For example, a provider attempting to prescribe diphenhydramine for allergic rhinitis received an evidence-based alert screen recommending fluticasone for this indication. In the original intervention, a provider wishing to change from diphenhydramine to fluticasone would have had to close out the alert screen, cancel the diphenhydramine prescription, and then begin the fluticasone prescription. With the one-click option, a provider was able instead to click a button that closed both the alert page and the diphenhydramine prescription, and automatically completed a weight-based fluticasone prescription. This one-click option was estimated to save each provider approximately 11 keystrokes or mouse clicks for each prescription dispensed.

Our second subanalysis studied whether or not the intervention effectiveness faded over time, even with continued alerts. To assess whether providers were tiring of the intervention, we divided the time following the beginning of the intervention into five quarters (3-mo periods) following the introduction of the intervention; within each time period, the intervention effect was otherwise assessed exactly as in the main analysis. Dividing the study in this way allowed us to see whether the effect waned over the period of the study, and to test whether providers might be paying less attention to the intervention with repeated exposure over time.

### Statistical Analysis

As in the original study, provider behavior change was measured as the difference, by study arm, between the outcomes in the period before and after the trial (except for bronchiolitis, as mentioned above). Measuring each provider's behavior as a change from baseline served two functions. First, it controlled for each provider's individual prescribing practices, and second, it reduced the random variance in the outcome measure, affording our analysis greater power. Because we measured the outcomes as a change in individual behavior, it was not necessary to control for provider-specific potential confounders. To test the primary hypothesis we used weighted regression analyses, controlled for clustering by provider, to test the difference in behavior change between the treatment and control groups. Weighted regression analyses, again controlling for clustering by provider, were also used to assess the statistical significance of the behavior change within the treatment and control groups.

The provider panels were unbalanced because of different work styles and schedules, and some providers had many more visits than others. As a result, the outcomes (mean change in provider behavior) were estimated with a greater degree of precision for providers with many visits than for providers with fewer visits. To account for this in the regression analyses, we conducted weighted analyses, whereby each provider's behavior contributed information to the analysis proportional to the precision with which their mean was estimated. As in the original analysis, this method achieved greater precision in the intervention estimates than unweighted analyses.

Not all providers treated patients for each randomized intervention during both the before and after period, and therefore, the number of providers contributing information to each intervention varied slightly.

All analyses were conducted with Stata, version 6.0, statistical software (Statacorp,http://www.stata.com).

#### Sample size.

The number of providers available to participate in this study was estimated a priori to be approximately 42. We calculated the power of the study adjusted for clustering effects using the method of Hayes and Bennett [38]. At the time the study was conceived, approximately 75% of otitis media cases were being treated with antibiotics, and we considered a clinically significant goal to be that of lowering this proportion to 60%—an absolute change of 15%. With 42 providers, we calculated that this treatment effect could be detected with 90% power with alpha = 0.05, assuming a standard deviation of 15% across providers.

## RESULTS

### General Descriptives

The intervention period lasted for a total of 50 mo at PCC (from November 1999 to December 2003) and for 18 mo at SP (from June 2002 to December 2003), during which 57,319 and 33,127 visits were made for pediatric care at the two sites, respectively. (The length of the intervention was longer at PCC since the network was already in place at PCC while it had to be constructed at SP.) At the two sites combined there were 1,933 prescriptions written for otitis media, 754 for acute sinusitis, 372 for allergic rhinitis, 235 for pharyngitis, 96 for croup, 85 for urticaria, 79 for bronchiolitis, and seven for constipation. The baseline rates of prescribing in accordance with the evidence are shown in Table 2; during baseline, the percent of prescriptions that were prescribed in accordance with the evidence were 38% and 39% in the intervention and control groups, respectively.

**Table 2:**
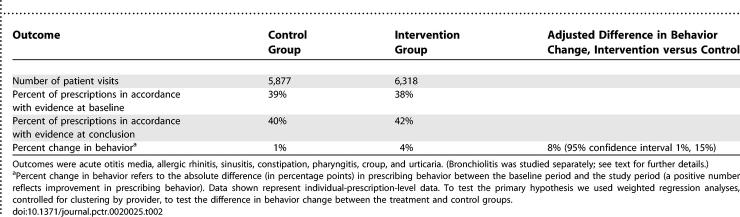
Summary of Behavior for All Outcomes across Sites

### Main Analyses

For the primary outcome of our study—all condition-specific outcomes assessed as a single group—the intervention had a statistically significant effect upon provider prescribing behavior (Table 2). Among providers not receiving the intervention, prescribing behavior in accordance with the evidence improved only marginally, by 1%, while among those receiving the intervention, prescribing behavior improved by 4%. The crude absolute difference in these behavior changes was 3%; the adjusted effect size was 8% (95% confidence interval 1%, 15%). This effect remained significant even after excluding the outcome of otitis media from PCC.

Looking at the conditions separately (Table 3), providers receiving the intervention at PCC showed statistically significant differences in the decision to treat otitis media and in their choice of medication for treatment of allergic rhinitis. They also showed improvements in the “combined” group of diagnoses, although this change was not statistically significant. At SP, the intervention group showed statistically significant improvements in the decision to treat otitis media. Interestingly, as we found in the original trial, at each site there was a secular trend towards increased treatment of otitis media with antibiotics, and our intervention served primarily to slow this trend among those providers receiving the evidence screens.

**Table 3:**
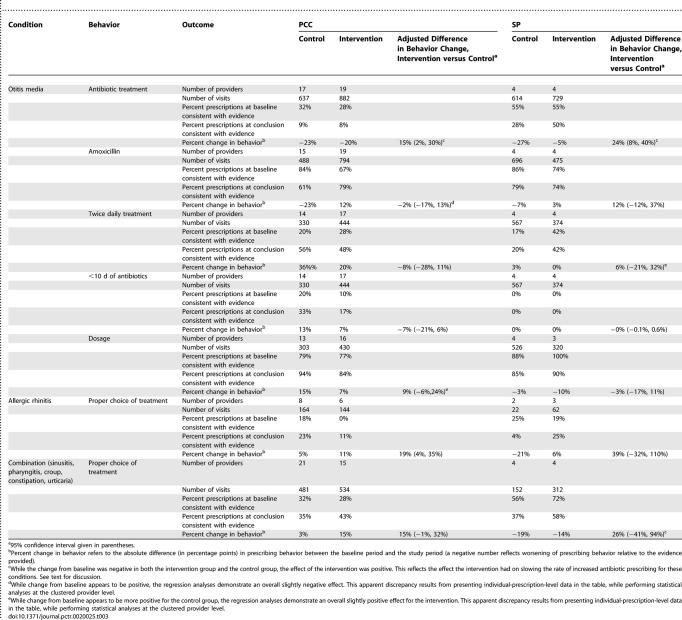
Summary of Behavior Changes for Individual Outcomes at Each Site

For bronchiolitis, providers in the intervention group at PCC prescribed albuterol for bronchiolitis substantially less often than providers in the control group (21% versus 32%, respectively; absolute difference of −11%; adjusted effect size of −6% [95% confidence interval −18%, 7%]). At SP the rate of prescribing for bronchiolitis was so low (one prescription for albuterol during the study period) as to preclude analysis.

### Subanalyses

In the one-click option subanalysis, there was little evidence that this option further impacted provider behavior. There was no significant change or improvement in prescribing for either otitis media or allergic rhinitis at either PCC or SP when the one-click option was added to the screens for providers already receiving the intervention. For example, at PCC prescribing amoxicillin twice daily for otitis media increased by 2%, while proper dosing for otitis media fell by 2%.

In the second subanalysis, we found little evidence of provider fatigue. For one condition—otitis media—the impact of the intervention was rather weak for the first two quarters of the intervention, but then demonstrated a markedly improved impact over the next three study quarters (9% less prescribing in the intervention group compared to the control group for otitis media in quarters 1 and 2, and 26%–27% less prescribing during quarters 3–5). When we looked at all conditions combined, the intervention demonstrated some variability but overall had a constant effect over the study.

## DISCUSSION

### Interpretation

In the Institute of Medicine report “Crossing the Quality Chasm” [1], the Committee on Quality of Health Care in America outlined a number of strategies for improving the quality of health care in the United States, including the redesign of care processes to reflect best practices and the use of information technologies to improve access to clinical information and to support clinical decision-making [8]. In our cluster randomized trial of using information technology to deliver evidence to pediatric providers at the time of prescription writing, we found an overall modest, yet statistically significant improvement in provider prescribing practices. The impact of the electronic prescription writer took some time (as opposed to having an immediate effect), and continued exposure to the message screens was necessary to maximize desired prescribing behaviors for some specific conditions such as otitis media.

There was considerable variation in the degree of effect of the intervention based on which message screen was being viewed. This is not surprising as the messages themselves differed in the strength of their recommendations. A key feature of the system (which distinguishes it from many previous interventions) was that the evidence-based support system was nether prescriptive nor proscriptive. The messages were developed from the best available evidence, which was frequently limited. In a few cases, the only evidence came from adolescent and adult studies, and providers had to decide if the study conclusions were applicable to children. Some of the messages included a “number needed to treat,” and providers likely weighed this information differently based on individual patient preferences, the condition being treated, and the severity of the condition at the time of presentation. This approach attempted to empower providers with information, and was in keeping with recommendations for applying evidence to practice [39–41], but it may have been less likely to alter behavior than a purely directive approach. While others have proposed using group consensus to create guidelines in the absence of clear treatment recommendations [42], we believe that this would have been counter to our intent to facilitate the use of evidence-based medicine. Another key element of our intervention was that it was integrated into practice in such a way as to present relevant information to providers at the point of care without disrupting workflow. Rapid access to information at the point of care may be key for changing provider behavior [32], but it is hard to accomplish in a busy clinical setting.

There were some limitations to our trial. First, only eight of the 44 providers we studied practiced outside of an academic training environment, limiting our ability to make inferences about the performance of this system in the general practice arena. Second, because of power limitations, we were unable to assess whether the evidence screens may have had different effects for clinicians at different levels of training. Finally, we specifically planned to study the ability of electronic point-of-care systems to effect physician behavior change, not the effectiveness of an electronic point-of-care system to deliver messages about a single condition. This required that, for our main outcome, we considered all behavior change to be of merit and of equal importance statistically. Such an assumption, while necessary for our study, might not necessarily be well founded. It may be that we would have observed stronger effects and more robust findings had we focused on only one or two outcomes and employed a more comprehensive and concentrated intervention geared towards effecting management change within these conditions. In this regard, future evaluations might focus on trying to effect behavior change for some particular conditions that, for example, drive a large proportion of health-care costs, or are associated with substantial need for prescribing improvement.

While for many conditions prescribing behavior improved, we found that antibiotic prescribing for otitis media increased for providers in both the intervention and controlgroups, counter to the evidence provided in the intervention. Our intervention served primarily to slow the tide towards increased prescribing. This increase in prescribing occurred even though recent publications included in the intervention evidence supported limiting antibiotic use [43,44], and the 1999 Washington State Department of Health's “Practice Guidance for the Judicious Use of Antibiotics in Otitis Media” [45] included a recommendation for observation and symptomatic treatment alone in patients with mild symptoms. We do not have a good explanation for this trend in behavior, although because we recorded only prescriptions written as opposed to prescriptions filled, it is possible that providers were writing some prescriptions but instructing patients to fill them only if their symptoms failed to improve without antibiotics.

Our subanalyses unveiled some important findings relevant to future implementation of these systems. We found that for some conditions there was a delay before the message screens achieved their fullest impact. Technological problems early in the study may have decreased early adoption of the system, or alternatively, providers may have needed time and repeated exposure to the evidence-based messages before they changed their behavior, especially behavior most ingrained such as for otitis media. The second subanalysis assessed our attempt to streamline electronic prescribing. Previous investigators have reported that ease, speed, and some control of the system is crucial to the successful adoption of a clinical support system [46–50], but we saw little impact from a one-click option that reduced keystrokes. This one-click option was introduced in the latter half of the study, and it may be that the intervention had already achieved its maximal effect in changing prescribing practices. In addition, providers already facile with the system and viewing message screens quickly could have failed to notice this additional feature when it appeared.

### Generalizability

An assessment of the generalizability of our findings is critical. One of our two sites was situated within a large academic medical center, while the other clinic was populated by many recent graduates of this same site. Additionally, the practices served a fairly well-educated and primarily English-speaking urban and semi-urban patient population. Hence, our findings might not be generalizable to pediatric practices that differ in meaningful ways either in terms of their patient demographics or provider practice patterns.

Additionally, the computer software that was developed and used for this trial was independent from any other computer system in place in these clinics, and was unattached to any other computerized messaging systems. Uptake of systems in other settings might indeed be different, especially if an entire suite of messages is provided that is designed to provide evidence and other information oriented towards improving delivery of care.

With regards to this last point, our findings are particularly pertinent to smaller clinical practices that might not be aligned with larger organizations, and who will have to choose EMR systems and DSSs on their own. During our trial we had a number of challenges related to computer software and hardware, and other studies have reported likewise [51,52]. For example, battery life for the wireless equipment was problematic, especially during the early days of the study, and numerous adjustments were required to solve this problem. Ideally, complex interventions should be first developed through an interactive process, and evaluations should be performed on stable systems [53,54]. However, this is easier said than done in the course of a clinical trial of a health technology, not just because of the practical time constraints of trial funding, but also because systems and technology continually change, improvements in software and hardware may cause other unforeseen, new problems, and the needs and wants of providers for particular features change over time. As electronic information systems become more widespread, rapid evaluation will be important in order to better understand the impact of electronic decision support on provider behavior [54].

We found that initial on-site training of all providers was insufficient to ensure ongoing use of the system, and use was not habitual until after providers had used the system for many months. We offered labor-intensive support with on-site promoters, research assistant presence, E-mail hints, and person-to-person rather than simply electronic feedback mechanisms—all among the recommended strategies for improving adoption of health technology applications [55,56].

Our effect size compares favorably to other considerably more intensive and expensive interventions [57–59] and has two distinct advantages. First, once the fixed costs of an EMR system are accounted for, the marginal costs of DSS are small. Second, if a stable system is in place, upgrades can be done to incorporate new evidence or other features.

### Overall Evidence

Any comparison of our findings to those of others must first recognize that there have been many different types of computerized medication management systems developed. Bennett and Glasziou [32] categorized these systems according to their type—provider reminders in outpatient settings, provider feedback, combined reminders and feedback, inpatient reminders, and patient directed reminders—and the intended setting, and presented a review of their effectiveness. Reminders differ from feedback in that the former refer to information delivered around the time of the encounter and directed towards a specific episode of care, while the latter refers primarily to aggregated information collected on patients or providers and delivered with the intent of affecting future clinical care decisions. Following Bennett and Glasziou's scheme, our trial is most comparable to a total of 11 other trials that have assessed provider reminders in outpatient settings. Of these 11, the evidence is quite divided in terms of showing effectiveness: five of the studies showed positive effects while six did not. Among the studies showing positive effects, three had relatively small effect sizes that were consistent with what we found in general. However, none of these 11 studies were performed on pediatric populations, making further inferences or comparisons problematic.

It is important to recognize that we studied only a part of the capabilities of this (or similar) systems. An 8% overall improvement in prescribing practices such as we found, along with other point-of-care interventions such as guidelines for chronic care, could have substantial long-term impacts, for example, on reducing antibiotic resistance or costs of care. Nevertheless, the challenge is to continue to improve the efficiencies of these systems. A recent study of computerized decision support in primary care found no effect of its guideline-based interventions due to low levels of use [60]. Given the current complexity of medical practice and the rapid pace of advancements in medical science, we believe that clinicians are overloaded with information and need these systems if their decision-making is to be evidence based [52,60]. Work is needed to provide better integrated, more robust, and more flexible products that meet providers' needs.

## SUPPORTING INFORMATION

CONSORT Checklist Click here for additional data file. (227 KB PDF)

Trial Protocol Click here for additional data file. (237 KB PDF)

Figure S1 Screenshot of Messages (38 KB PPT)

## Abbreviations

CPOE: computerized provider order entry
DSS: decision support system
EMR: electronic medical record
HCP: health-care provider
PCC: Pediatric Care Center at the University of Washington
SP: Skagit Pediatrics
