# Supplementary material for: A Cluster Randomized Clinical Trial to Improve Prescribing Patterns in Ambulatory Pediatrics
Source: PLoS Clin Trials. 2007 May 18;2(5):e25. doi: 10.1371/journal.pctr.0020025 (PMC1876598; doi:10.1371/journal.pctr.0020025)
Supplement: Trial Protocol [file pctr.0020025.sd002.doc]

### Pediatric Evidence Based Medicine – Getting Evidence Used at the Point of Care

## Section A. Overview & Specific Aims

The main goal of our study is to improve the dissemination of evidence at the point of care for pediatric patients, and thereby increase the application of evidence-based medicine to child health, change physician behavior, and expedite the translation of research into clinical practice. To do this, we intend to conduct a randomized controlled trial to address two main study hypotheses.

Study hypothesis one: Use of an evidence-based decision support system at the point of care will improve antibiotic and pharmaceutical use in acute outpatient pediatric care. Specifically the decision support system will (i) reduce the duration and frequency of antibiotic therapy for otitis media, (ii) reduce the duration of antibiotic therapy for acute sinusitis, (iii) increase the use of less expensive antibiotics such as amoxicillin, where appropriate (iv) reduce the use of bronchodilators in the outpatient treatment of bronchiolitis, and (v) increase the use of intranasal steroids for allergic rhinitis.

Study hypothesis two: Individualized physician feedback, triggered by the decision support system and delivered in a manner intended to reinforce physician behavior change, will provide additional benefit for appropriate pediatric drug utilization when used in conjunction with the evidence-based decision support system.

# Section B. Background, Rationale and Significance

## B.1 Child Health Service Research

Research on children’s health services lags behind those of other disciplines (Forrest 1997). This state of affairs is the result of relatively low funding for children’s health research, a paucity (until recently) of pediatric health services researchers, the low cost of child health care compared to adult and elderly populations, and the predominance of disease orientation rather than child-specific issues in health services research. In addition, while appropriate laboratories exist for adult and elderly medical issues, these same facilities do not exist for children. In a review of interventions designed to change test ordering behavior, 33 of the 49 articles outlined in tabular form excluded pediatrics, and – while some included family physicians or emergency department physicians – *none* of the interventions on behavior change focused specifically on pediatrics or child health issues (Solomon 1998). A call has gone out for the development of child health services capability in appropriate laboratories. These include much needed linkages between academic centers and clinical practice sites.

## B.2 Efforts to change physician behavior

Health care providers are under considerable pressure to find a balance between doing what is best for their patients while also making health care more efficient and cost-effective. In addition, physicians, medical organizations, and national organizations are increasingly aware of the long lag that exists from the time research shows benefits of treatments and their subsequent widespread utilization in the clinical realm (Antman 1992). Physicians and others within the health care system are being called upon to improve the extent and speed with which research or quality improvement efforts are implemented into practice. Attempts to expedite change have included continuing medical education, peer review with feedback, academic detailing, administrative interventions, clinical practice guidelines, and financial incentives (Greco 1993). Unfortunately, most of these interventions have not been particularly effective, and many have been costly to implement or difficult to maintain. Administrative interventions including pathways, reminder systems, and computer decision support have achieved more success at changing physician behavior (Oxman 1995).

The PRECEDE model for behavior change presents a conceptual framework for planning interventions to promote long-term physician behavior change. The steps for physician behavior change can be broken down into three stages: predisposing, enabling, and reinforcing factors. Predisposing factors include attitudes, knowledge, and beliefs that make physicians (or patients) more likely to accept certain types of behavior change. Enabling factors specifically focus on the resources or structural systems that serve to facilitate (or prevent) certain types of behavior. Finally, reinforcing factors serve to reward (or extinguish) certain types or patterns of behavior, primarily by means of feedback. The PRECEDE model outlines the key components that are instrumental to large scale interventions focusing on permanent physician (or institutional) behavior change.

In a review of interventions aimed at changing test ordering behavior among physicians, Solomon showed that the more successful interventions were those that incorporated a multimodal approach, especially those with a combination of predisposing, enabling, or reinforcing factors (Solomon 1998). Audits, continuous quality improvement, and administrative interventions appeared to be the most successful. However, much more needs to be learned about how to create long-term solutions to change physician behavior in an on-going manner. Specifically, in order to promote long term change and perpetuate desired behaviors in a medical environment where ‘correct’ evidence changes constantly, we need to design and evaluate systems that integrate evidence into the point of care, and therefore enable the provider to provide appropriate care.

## B.3 Computer decision support systems

Computer decision support systems can improve physicians’ appropriate use of antibiotics (Pestotnik 1996; Evans 1998; Bates 1998; Raschke 1998). Although computer-assisted decision support is not new, it is still undergoing rapid development concomitant with advances in computer technology (Johnson 1995). Currently, many current decision support systems are hampered by two limitations. First, the systems frequently require that physicians actively engage the system in their search for relevant information. That is, busy clinicians must divert their attention from the immediate task at hand—treating a patient—to access an information system (Shortliffe 1989). Even when such systems are available and within easy reach, they can be time-consuming and can only affect change in situations where clinicians actually seek help. Many daily decisions (e.g. the prescribing of antibiotics) are not based on current evidence (Nyquist 1998; Pennie 1998) because clinicians are unlikely to seek new or more recent evidence due to the extra steps involved, and their lack of skill in critical appraisal of the medical literature. Second, some decision support systems simply provide too much information for busy clinicians to process. Automated World Wide Web or MEDLINE search engines represent remarkable innovations in computer technology but can inundate providers with information. Thus, when a provider has a specific question in mind (e.g. should I treat otitis media with effusion with antibiotics), they are referred instead to an entire guideline on-line, or referred to dozens of articles on MEDLINE rather than being given the short or pertinent answer.

A common concern in the application of evidence-based medicine has been that there is not enough evidence to support common medical care decisions. However, we believe that this is no longer the rate limiting step. The evidence reviews produced by the AHCPR funded centers, the Cochrane Collaboration, the York Center, and those increasingly published by peer reviewed journals are rapidly filling the knowledge gap of ‘what works’. The challenge now is to apply this information at the point of care. Unless the information is presented in a usable manner (one that is rapidly assimilated) and without the ‘request’ of the provider, it will not be used.

Our project will focus on developing innovative ways to deliver information and evidence at the point of pediatric care. We have developed an Evidence Based Decision Support System (EBDSS), designed to provide relevant yet brief bits of evidence at the moment of key diagnostic and therapeutic decisions. While this “just in time” mode has been shown to be effective, it has not been assessed in busy general pediatric practices, nor in a diverse array of practice venues such as we propose (Chueh 1997).

Our project will build on the efforts of others that are already systematically reviewing the efficacy of common medical practices. These reviews – such as those produced by the Cochrane Collaboration or the AHCPR – are extensive assessments and evaluations of best treatments and practices.However, these products can be difficult for providers to digest quickly and apply to patients on daily basis (Christakis 1998). In our project, all prescriptions will be done ‘on-line’ using computers in examination rooms. We will use this infrastructure to intercept and guide physicians, using evidence delivered at the point of care. To avoid information overload, we will use the EBDSS to provide the evidence in stages. The first stage will be a four to five ‘bullet’ sentence summary of the most relevant and compelling point (including the clinical bottom line e.g. “No antibiotics have been shown to be more effective than Amoxicillin for first line treatment for otitis media”.) At the bottom of this screen there will be three option buttons (1) “Tell me more” which gives the next level of evidence, e.g. the summary odds ratios comparing responses to amoxicillin vs. other antibiotics (2) “Show me the reference” which produces on screen a meta-analysis (from which the recommendation came) that has been scanned onto the local network hard-drive (3) “e-mail me the reference” which sends the reference via e-mail to the provider for their review later. An example of these staged screens is shown in Appendix B.

## B.4 Significance of the proposed project

Our intervention builds on several features of systems shown to be effective at changing physician behavior, adapting some proven methods and evaluating some new ones. First, our research focuses exclusively on the medical care of ambulatory pediatric patients who have heretofore not been the focus of much research in physician behavior change. Second, by providing evidence to support or contradict certain prescribing practices, we have adapted the education outreach model which has been shown to be effective (Avorn 1983). Our hypothesis is that the system's affiliation with the U of WA Department of Pediatrics will lend sufficient credibility to the evidence presented that it can function as "cyber academic detailing." However, our approach is considerably less labor intensive and therefore may be more cost-effective than in person detailing. Third, in ways analogous to reminder systems that have been shown to improve the provision of preventive care services, our system aims to provide real time reminders for other clinical services that pertain to acute care. Fourth, although audit and feedback have been shown to be effective, the cycle time employed previously has been more than 30 days. Our system will provide feedback within a week of actions. Fifth, use of our EBDSS will not be optional nor will it require effort on the part of providers. Sixth, our system will not restrict medication use and won’t impinge on physician autonomy. Finally, our system provides real time access to the primary sources from which the evidence was derived.

The unique aspects of our study are outlined in the table below:

Innovative system aspects

| ***Prior Work*** | ***Proposed Study*** |
| --- | --- |
| Almost exclusively adult inpatient study populations | Ambulatory pediatric study population |
| Generic computer based reminder systems (Bero 1998; Johnston 1994; Hunt 1998) | Context sensitive real-time reminders |
| In person academic detailing (Avorn 1983) | Academic detailing via computer |
| Reminders or feedback in person or via regular mail | Reminders and feedback delivered electronically |
| Long cycle times (>30 days) for provider feedback | Short cycle times (<7 days) for provider feedback |
| Optional use of EBDSS | Mandatory use of EBDSS |
| System restricts therapy choice | No restriction on choice of therapy |
| No real-time access to source literature | Real-time access to source literature without the need for literature searching |
| Guideline based | Evidence based |

# B. 5 Cost implications of the intervention

While there has been considerable interest in the clinical implications of the use of decision-support systems (both evidence-based and guidelines-based), there have been few formal studies of the cost implications of their use. As with any intervention, the effectiveness and appeal of the EBDSS will be limited if costs are prohibitive relative to the benefits.

This study therefore proposes to complement the clinical trial with a cost analysis of the intervention. Because the interest of the study is primarily process-oriented, with only a modest expected impact on clinical outcomes, the cost analysis will take the form of a cost-minimization study. The purpose of this study will be to determine whether the intervention can potentially pay for itself by introducing cost savings in excess of program costs.

The study will compare the costs of the intervention (as estimated for a real-world setting) to potential cost offsets from reduced resource use. Cost offsets will include reductions in the prescription of drugs, and in office visits related to side effects of antibiotic use. Potential additional cost-offsets will be longer term, and would include reduced visits as patients acquire knowledge about treatment efficacy, increased efficiency and reduced danger from illegible prescription forms, and more complete billing for procedures and services actually provided. The methods of the cost minimization study are discussed in greater detail in section D.7.d.

**Section C. Preliminary Studies**

Our team at the University of Washington and Children’s Hospital and Regional Medical Center has been involved with extensive efforts to translate research findings into changes in practice. The proposed program builds on our current and past activities. In this section, we will review those activities which are directly relevant to the proposed project.

**C.1 Training of faculty, residents and students in pediatric evidence based medicine**

In 1997, we received funding from the Culpepper Foundation and the David and Lucille Packard Foundation to train faculty, residents and students in the principles and practices of pediatric evidenced-based medicine. This program consisted of a number of components.

We hired two fellows to serve as resources for faculty and residents on the general pediatric wards at Children’s Hospital and in continuity clinic sites throughout our training program. The fellows led journal clubs, gave didactic lectures, and attended inpatient work rounds. On rounds and in the clinics, the fellows concentrated on helping residents and faculty ask appropriate searchable questions, helped them to conduct efficient literature searches to identify evidence, and helped them to critically appraise the literature. The most commonly examined topics were diagnostic and therapeutic issues that arose on the wards and in the clinics: steroids and bronchodilators in the treatment of bronchiolitis; high dose albuterol and the need to monitor serum potassium concentration in asthma; diagnostic radiology in the evaluation of children with urinary tract infections; the diagnostic utility of dipstick vs. full urinalysis for UTIs; the need to treat every episode of otitis media with antibiotics; and the utility of diagnostic radiology in acute sinusitis. The fellows focused on the Cochrane database or searched for relevant systematic reviews or meta-analyses. In many cases there was only a single randomized trial. These articles were then summarized and the answer brought back to the clinical team. While others have suggested that these types of queries can be accomplished quickly and in ‘real time’, our experience suggested that the average length of time to produce each CAT (Critically Appraised Topic) ranged from 2-7 days. In some cases, there was no published data relevant to the question, and this result too was also brought back to the clinical team.

In addition, we carried out the following activities:

- A faculty meeting was held to discuss resident teaching and ways to incorporate EBM into faculty practice and teaching. All 130 pediatric faculty were given a copy of the handbook, Teaching Evidence Based Medicine by Sackett et al.
- A pediatric Grand Rounds was devoted to the uses of EBM in pediatrics.
- Resident journal clubs were organized to teach the principles of EBM. The “Users’s Guide to the Medical Literature” series in JAMA on critical appraisal of the literature was used as the basis for these sessions.
- Residents in their second year used to be required to give a talk as part of their ambulatory rotation; this was changed to be an evidence-based review on a particular topic.
- Dr. Jack Sinclair from the Department of Pediatrics, McMaster’s University held a one day workshop with the entire faculty and fellows of the Division of General Pediatrics on pediatric EBM. Community physicians serving as resident preceptors for continuity clinic also attended this.
- Five faculty and a pediatric chief resident attended the week long workshop on teaching EBM sponsored by McMaster University.

**C.2 Assimilation and dissemination of evidence on pediatric health care**

In conjunction with the training outlined, evidence on the topics cited above was assembled in a common format, that of the short Critically Appraised Topic or CAT. In order to make this information widely available to faculty, residents and students, we created a web site for the dissemination of this information (http://depts.washington.edu/pedebm/). This web site provided links to useful EBM related sites around the world, with a special focus on pediatric sites.

The Critically Appraised Topics were further distributed by our Electronic Morning Report (EMR) – an email forum devoted to discussions of clinical issues. The EMR is distributed to affiliate faculty and to graduates of the program, and in the past two years we have received comments from graduates in other states and from clinicians in the United Kingdom.

In addition to these projects, we have continued to assimilate evidence for use in the evidence-based decision support system described below. We have hired an MPH epidemiologist to search for evidence on specific topics, to synthesize this evidence, and then to present it in readily available and accessible formats.

At present, computer work stations with access to the web and to Medline have been placed in all physician work areas in Children’s Hospital and in the clinics at Harborview Medical Center and the University of Washington Primary Care Center. This has allowed easy access to EBM resources, including the Cochrane Library.

**C.3 Evidence Based Decision Support System at UWMC**

The Evidence-Based Decision Support System (EBDSS) at the Pediatric Clinic in the University of Washington Primary Care Center (UW) was developed after our experience attempting to have residents and other physicians employ EBM in their daily care of patients. We found that providing fellows to teach EBM and a website devoted to EBM may have increased physician knowledge and changed attitudes, but did little to actually alter physician behavior or affect patient outcomes. We found, as have others, that translation of this information into practice was inconsistent and irregular (Grol 1998).

Therefore, over the last year we have developed an online EBDSS in order to provide evidence in real time, without its being ‘requested’. The basic tenet has been to “intercept” the physician during critical moments of patient care, while they are ordering prescriptions. By doing this we hope to reduce the constraint of providing new evidence only through opinion leaders, academic detailing, grand rounds, journal clubs, or other traditional teaching methods. Moreover, this method obviates the need for physicians to seek out evidence: it has been brought to them without any extra effort on their part. Using this infrastructure, new developments can be introduced very rapidly and can in effect be instantaneously brought to the attention of the provider.

The current system has been implemented to date for prescription medication ordering in the Pediatric Clinic at the University of Washington Primary Care Center. Computers were purchased for each of the 13 exam rooms and networked with Windows NT to a dedicated server. The pharmacy formulary for the UW Medical Center was downloaded onto our clinic network.

At present, all providers use computers provided in the examination rooms to write prescriptions (although not part of this study, we will soon be extending this to include diagnostic tests). For a prescription, the provider clicks on the “Rx” icon and a prescription blank appears with the patient’s name, date of birth, medical record number and weight already recorded. This is automatically captured into the system from the nurse and reception registration data. The provider is presented with a list of the most commonly used medications in a pop-up menu (Screen 1, Appendix A). A drug is selected, and the indication (e.g. otitis media, streptococcal pharyngitis, UTI) is specified (Screen 2, Appendix A). The provider selects the dosage, duration, and regimen for each prescription, which is built on the screen as each step is completed by the provider (Screen 3; Appendix A). For many medications, such as acetaminophen, automatic dosing is available; the quantity to dispense and the dose are automatically added. The provider can over-ride the calculation by selecting any field on the prescription form and typing the information directly into the prescription. Finally, the prescription is printed, including the provider’s name, billing number and controlled substance number (Screen 4; Appendix A). At the current time residents at our main clinic are using the system (including the automated features described), but we have not introduced the ‘evidence’ that will comprise our intervention.

The system, behind the screen, automatically captures data on provider ID, indication for medication, choice of medication, dose and duration prescribed. This information is captured on a Paradox Program and can be downloaded in an ASCII file, manipulated with ACCESS or other database programs, and analyzed with standard statistical packages such as Stata, SAS or SPSS.

The prompts that are presented to physicians are simultaneously condition and treatment specific. An example is given to demonstrate how these prompts work in real-time: Currently a physician diagnoses a case of otitis media, and elects to start the child on amoxicillin. He/she ‘clicks’ the treatment (Rx) button, and is presented with an on-screen list of the formulary, in alphabetic order. He/she clicks on ‘Amoxicillin’ and is then presented with a pop-up screen listing the most common indications (i.e. otitis media, sinusitis or phayngitis) He/she then clicks on the indication, dosage options (20 mg/kg; 30 mg/kg/40 mg/kg; 50 mg/kg; other), and duration (5 days, 7 days, 10 days, 21 days, Once the provider clicks on duration, the prescription is automatically calculated, filled in, and printed out. Although this process may sound cumbersome, our experience to date has shown both that (i) using the EBDSS saves between 10 and 30 seconds per prescription compared to hand written prescriptions, and (ii) physicians are pleased with, and accept the EBDSS as part of their daily practice.

Our intervention (as separate from the EBDSS as it currently operates) adds a single screen to this process. In this example, after the provider clicks on ‘otitis media’ (to specify indication), he/she is presented with another screen that comes up side-by-side to the EBDSS. This screen shows the following evidence:

- AOM otalgia resolves within 7 days in 86% of children given analgesics only
- AOM otalgia resolves within 7 days in 90% of children treated with antibiotics (& analgesics)
- 22 children would need to be treated with antibiotics to relieve one additional case of otalgia within 7 days
- For every case of otalgia relieved, 1-3 children will experience diarrhea as a side effect of treatment

There are 2 additional buttons at the bottom of the screen that state “To read more about this, click here”, and “To see the abstract, click here”. In Appendix B, we show the screens that a provider would see if they clicked either of these buttons.

The system currently has a great deal of flexibility because each drug can be built for specific indications, and dosages and forms can be linked to specific indications. While we can create these for a large number of medications (including all those on the University of Washington formulary), for the purposes of this study, we will be studying only a small number of specific conditions listed previously.

This decision support system is currently operable at the Pediatric Clinic in the University of Washington Primary Care Center. It runs in Windows NT via network server at the center, and is currently independent of the electronic medical record. Therefore, the decision support system does not rely on capturing any data from the medical record. The clinic nurse enters the patient’s name, current weight, birth date, and medical record number at the time of the visit. At other sites, the decision support system will run on Windows operating systems, and will not require integration with other electronic medical record software.

A survey of physicians at the Pediatric Clinic at the University of Washington Primary Care Center revealed that the system has been well accepted and perceived as adding value. We performed an informal survey of the providers 3 weeks after they began using the EBDSS. To the statement “I use the prescription writer”, 25% of respondents replied “All the time”, while 60% stated “Most of the time” and 10% said “Rarely”. Only one provider (5%) stated He/she “Never” uses the prescription writer. The following responses were also obtained:

|  | Yes | No | Not sure |
| --- | --- | --- | --- |
| The Rx writer is helpful to me now | 14 (70%) | 1(5%) | 5 (25%) |
| The Rx writer has the potential to be very helpful to me in the future | 15(75%) | 1(5%) | 4 (20%) |
| The Rx writer saves me time | 12 (60%) | 3 (15%) | 5 (25%) |
| I prefer the Rx writer to manual prescription writing | 17 (85%) | 2 (10%) | 1 (5%) |
| The Rx writer can improve the care of my patients | 12 (60%) | 3 (15%) | 5 (25%) |

It is important to understand that these early results were obtained during the beta-testing of the EBDSS. We have since eliminated the pads for written prescriptions, so that all prescribing is currently done ‘on-line’ with the computer at the University of Washington Pediatric Clinic. The response to this changeover has been very positive, and we have not encountered resistance to this format for writing prescriptions. To improve our understanding of physician’s attitudes and beliefs regarding the EBDSS, we will survey them again at 6 and 12 months into the study. A copy of the survey instrument is shown in Appendix C.

This decision support system will serve as the tool for incorporating evidence into clinical care, as described in Section D.

**C.5 Systematic reviews and other EBM activities of investigators**

The investigators on this project have had considerable experience with other EBM related activities.

- Dr. Davis has been a faculty member of the pediatric EBM course sponsored by the Institute for Child Health in London.
- Dr. Davis is leading a review group for the US Preventive Service Task Force on utility of neonatal hearing screening.
- Dr. Rivara is associate editor of the Cochrane Injury Review Group and has contributed one review to the Cochrane library and has another in progress. He also was editor of a special supplement in January 1999 to the American Journal of Preventive Medicine on motor vehicle injury prevention strategies, and is currently coordinating systematic reviews for the CDC on occupational related injuries. He was editor and director of a project conducting systematic reviews of child injury prevention strategies.
- Dr. Christakis conducted a national survey of pediatricians to determine the usefulness of guidelines for practicing pediatricians (Christakis 1998). He found that guidelines are not commonly utilized, and are not viewed as being helpful in their present format. We interpret this to mean that evidence must be presented in a different way to physicians in order to be utilized.
- Drs. Davis and Christakis led a 2-day workshop on Evidence- Based Medicine at the University of Washington in Fall, 1998. Participants included faculty and fellows from a wide range of disciplines at the U.W, as well as visitors from University of Michigan and University of California, San Diego
- **C.6 EBM activities in other departments at the UW relevant to this proposal** Dr. Davis has been the principal investigator on three studies at Group Health Cooperative of Puget Sound, both funded by the Centers for Disease Prevention and Control. The first study looked at the implementation of the new CDC peri-natal Group B Streptococcal prevention guidelines. The study assessed how well managed care organizations were able to implement system-wide changes in pregnancy management of GBS, as well as barriers to guideline implementation. The second study looked at the implementation of the new polio vaccination schedule at GHC and Northern California Kaiser, and how this affected immunization status among children in both managed care organizations. The third study used a randomized clinical trial design to assess methods of reducing overall antibiotic usage, and improving appropriate antibiotic use, among physicians at Group Health Cooperative and at Harvard Pilgrim Health Plan in Boston, Massachusetts.

## Dr. Christakis is the principal investigator on a study to determine whether a clinical reminder system, connected in real time to a statewide immunization registry (the Washington State CHILD registry) can significantly improve immunization rates in an inner-city pediatric clinic.

## Section D. Methods

# D.1 Overview

The main study design for this project will be a series of randomized controlled trial interventions designed to study the effect of introducing evidence at the point of care. Our method of delivering the intervention will be the computerized EBDSS, described above. The system will function on-line, in real-time during the patient encounter, and will be our method to provide current evidence to physicians and patients at the point of care.

In this current project, we will change the method of medication such that it will be entirely computerized within each examination room within our clinic study sites, as we have already done at the Pediatric Clinic at the University of Washington Primary Care Center. Using these computers, we propose to provide evidence directly to the provider (and/or the patient, as appropriate) at the point of care and at the moment that a prescription is written. After having physicians indicate (interactively on-line) the indication for antibiotic use we will use instantaneous pop-up screens to provide evidence pertinent to the decision to use a specific medication.

Additionally, we will study how automated electronic feedback, provided to some of the physicians using the EBDSS, works to reinforce appropriate physician behavior and maintain momentum.

## D.2 Intervention Sites

We will introduce our intervention at 3 separate clinical sites, in order to assess the generalizability of the intervention. The sites for the intervention will be

- An academic university pediatric clinic (known as the Pediatric Clinic at the University of Washington Primary Care Center (UW)). This clinic is affiliated with the University of Washington Division of General Pediatrics, with an average of 8.5 FTE physicians, residents and pediatric nurse practitioners working daily. It houses 12 exam rooms, sees approximately 12,500 visits per year and produces 200-300 written prescriptions per month.
- An academic university family medicine clinic (the Family Medicine Clinic at the University of Washington Primary Care Center). This clinic is operated by the University of Washington Department of Family Medicine, with an average of 12.2 FTE physicians, residents and physician assistants working daily. It houses 20 exam rooms and sees approximately 3,200 pediatric visits per year.
- A semi-rural private pediatric practice, Skagit Pediatrics, located in Mount Vernon, Washington. They have up to 5 providers (pediatricians and nurse practitioners) working daily in 4 exam rooms. They see approximately 16,400 pediatric visits per year.

## D.3 Study Methods and Analytic Framework

**D.3.a Overview of intervention:**

Each intervention will be introduced via a set of standardized information modules delivered on-screen to the provider at the point of care. This information module will look similar regardless of the subject manner. There will be 5 ‘bites’ of information delivered in each initial screen. For antibiotics and other therapeutics, this information will follow the format of: (1) the expected resolution rate in untreated patients (the natural history of the disease); (2) the expected resolution rate in treated patients; (3) the number needed to treat (NNT); (4) the number of patients with treatment related side-effects per number needed to treat; (5) a very brief summary (1-2 sentences maximum) of a pertinent guideline, systematic review, or meta-analysis.

Our expectation here is two-fold. First, by delivering evidence in a standard format, providers will quickly become adept at digesting and synthesizing evidence and applying it to patient care. Second, we have found that parents and other caregivers are receptive of this amount of information, and we intend to have this used to promote shared decision making between parents and medical providers. This information can be printed directly and handed out to parents or other caregivers.

At the end of this initial screen, there will be a link that says “Tell me more” ( 2). This will link directly to the “CAT”: the critical appraisal that we prepared for this topic. At the end of this second screen, there will be another link that says “Show me the reference”, and this will link to the relevant guideline, meta-analysis, systematic review, or randomized trial that supplied the information used in the first screen. These CATs and publications have been scanned into our network, and hence access to them is instantaneous. (This will also be loaded onto the computers at the other sites). If requested, these CATs and other publications can be printed directly from the workstation (the printer typically sits at the nursing station, but this varies slightly by site). A final button “e-mail me the reference” allows the provider to build in their own feedback, and to request that the reference or evidence be emailed to them at a later date for their review.

Our expectation with these sequential screens is not that the provider will interrupt a busy clinic day to read one of the CATs or systematic reviews while in the exam room. Rather, we expect that the physician will likely go back to this part of the EBDSS during a slow point in the day (or at the end of the day) and read further. However, we do expect that our intervention (the evidence on pop-up screens) will be used by providers at the time of the patient encounter.

**D.3.b.1 Main intervention, randomization:**

We will assess the effect of each intervention by means of a series of randomized clinical trials. For each intervention, at each of the three specific clinical sites (the two University clinics and the Skagit clinic) physicians will be randomized to receipt or non-receipt of the EBDSS and feedback intervention. (A sub-study that will allow analysis of the independent effect of the EBDSS and feedback separately, in described below is D.3.C.2).

The randomization of individual providers will take place in 2 stages. In the first stage, we will collect baseline data on practice patterns of individual physicians. This will include specific data on practice habits including antibiotic usage. This assessment will be completed during the first 3-6 months of study. At the end of this three - six month baseline period (depending on each site), we will analyze the baseline data and categorize providers at each site with regards to their antibiotic usage behavior. This categorization will then be used for randomization.

We will use a stratified and blocked randomization scheme: within each clinic site, providers will be stratified by antibiotic usage and randomized in blocks of two. For example, at Skagit Pediatrics we will take a block of 2 providers with roughly equivalent antibiotic use patterns. One of them will receive the intervention while – by definition – the other will not.. The result of this stratified and blocked randomization scheme is that (a) roughly equivalent numbers of providers within each site will be randomized to each arm of the intervention (maximizing study power and efficiency) and (b) there will be control for baseline antibiotic usage patterns since these will now be distributed equally among providers receiving (and not receiving) the intervention. There are approximately 85-90 providers from all 3 sites who will be randomized as part of this study.

This randomization process is shown in the flowchart.

It is important to note that each provider will be randomly assigned to receive only some of the evidence modules (the EBDSS and feedback intervention group), and may serve as the control for others (the current practice group). For example, a provider at the Pediatric Clinic at the University of Washington Primary Care Center may be randomly assigned to receive the evidence modules for otitis media and for allergic rhinitis, but then may also serve as a control physician for the interventions of sinusitis and bronchiolitis. (The intervention topics are outlined more fully below). A considerable amount of additional information is available on the computers in each examination room (such as the entire U.W. formulary), and it is unlikely that providers will even realize that they are not receiving some of the evidence modules.

## D.3.b.2 Main intervention, introduction of evidence

As mentioned earlier, the baseline period will be used to collect information on antibiotic usage patterns for provider randomization purposes. Screens for all four of the pharmaceutical interventions have already been developed. After randomization of the providers, the intervention modules will be accessible to those providers selected to receive the intervention.

These interventions will be introduced within the first 6-12 months at the UW Family Medicine clinic and at Skagit Pediatrics. Because the introduction of the interventions is staggered somewhat, the baseline data collection time period will take place over 6 to 18 months, depending on the intervention. The data collection following the introduction of evidence will vary from 12 to 24 months. This duration of data collection will allow us to measure both the short term as well as long term effects of the interventions.

The evidence will be ‘uploaded and turned on’ in the EBDSS for these topics at the appropriate time within each clinic. A programmer will spend a series of visits at each site to make sure the EBDSS is loaded properly and is providing the information in the intended manner.

**D.3.c.1 Secondary intervention:**

For each of the interventions, we will also incorporate a secondary intervention: that of physician feedback triggered by the support system, so that providers will receive electronic mail feedback as part of the intervention

This electronic feedback mail will be triggered when the provider accesses the decision rule, and will arrive in their e-mail 1-2 days after their use of the rule. The message will be brief, and will encompass salient evidence- based messages, such as: “You recently ordered antibiotics for one of your patients with otitis media. On the ordering system we provided you with evidence suggesting that 5 days of antibiotic therapy for otitis media is equivalent to 10 days of treatment. More judicious use of antibiotics will help to combat the growing problem of antibiotic resistance.” Additionally, those residents and faculty randomized to the feedback arm will also receive quarterly reports by email detailing the antibiotic usage patterns for otitis media within the entire clinic, and how their own antibiotic usage compares to that of the overall pattern within the clinic.

This type of electronic feedback will incorporate two different important mechanisms known to influence physician behavior. First, rapid feedback (within 1-2 days) of the patient encounter will likely reinforce provider knowledge of the evidence- based decision rule. Second, the individualized nature of the feedback (a short message from the study investigators along with quarterly reports of antibiotic use), is a form of computerized ‘academic detailing’, which has been shown to be an effective method of enhancing behavior change.

**D.3.c.2 Assessment of primary and secondary interventions separately, using a factorial design sub-analysis**

For the most common disease that we intend to study – otitis media – we expect to have a sufficient number of events in order to do an assessment of the additional effect of physician feedback when added to the EBDSS. For this particular intervention, the randomization scheme will be slightly more complex, and will follow a 2x2 factorial design. Providers will be randomized to one of four interventions (rather than receipt of intervention vs. not): Group 1: receipt of EBDSS and electronic feedback; Group 2: receipt of EBDSS only; Group 3: receipt of electronic feedback only; and Group 4: neither EBDSS nor electronic feedback.

The purpose of this particular intervention will be to allow us to study the following three questions: (1) what is the magnitude of the effect of electronic feedback or EBDSS when these are introduced separately from each other; (2) is there any ‘added benefit’ of the EBDSS once electronic feedback is in place; and (3) is there any ‘added benefit’ of electronic feedback, once an EBDSS is in place. This sub-analysis will also allow us to address a separate question of whether any effect we see is due to the education component (in which case electronic feedback might be expected to have the same effect as the EBDSS) or whether the effect is due to the provision of this information at the point of care (in which case the EBDSS would be expected to have a larger effect compared to the electronic feedback component.)

## D.4 Rationale for study design

A randomized clinical trial will be the strongest study design available to assess the effect of the EBDSS upon provider behavior, since it will directly compare providers receiving the intervention to those not receiving the intervention. Because the randomization will occur within sites, and will be stratified by baseline prescribing behavior, there will likely be good control of these important potential confounding variables.

We think that it is feasible to have randomization within a site, even though there is the potential for contamination between providers receiving the intervention and those not receiving the intervention. Direct contamination is likely to be minimal: the EBDSS and the electronic mail reminders are likely to be privately viewed messages that only briefly engage the practitioner and their patients. Additionally, even if providers do discuss the EBDSS and/or the automated feedback features, those providers randomized to not receive the EBDSS or feedback will not be able to access this feedback. It is important to realize that any particular provider will be randomly assigned to the intervention for many of the outcomes under study, and will probably remain unaware that –while they are receiving pop-up screens detailing evidence for some of the conditions - they are not receiving others. Hence we think the likelihood for serious cross-contamination for this part of the intervention is low. Nevertheless, using a before/after design in one of our sub-analyses, we do intend to study how evidence might disseminate even when a provider is randomized to not receive the evidence. This will give us valuable information about how future dissemination approaches might be planned. The analytic plan for this phase of the analysis is specified in D.7.c.

##### D.5 Topics

For our intervention we are concentrating on therapeutic decisions in six common diseases.. The topics were chosen based on (1) an analysis of the most common diagnoses seen our Pediatric Center; (2) those topics for which there exists substantial variation in care, and (3) those for which evidence is available and relatively clear.

**D.5.a Topics for interventions on antimicrobial use**

- *Acute Otitis Media:* Acute otitis media is one of the most frequent diseases in early infancy and childhood with an incidence rate of 0.93 episodes per child per year (95%CI 0.90 to 0.96) by 24 months of age (Alho 1991). It represents the second most common reason antibiotics are dispensed to children under the age of 5 (McCaig 1995). It has a high morbidity and low mortality (Stool 1989). Approximately 10% of children have an episode of acute otitis media by 3 months of age. The peak age-specific incidence is between 6 and 15 months (Klein 1989). Despite a large number of published clinical trials, there is considerable variation in the therapy of acute otitis media. We will address two aspects of treatment of acute otitis media: whether antibiotics should be used at all, and the duration of antibiotic use.

A meta-analysis by Del Mar indicates that early treatment with antibiotics compared to placebo does not reduce the risk of pain at 24 hours (OR 1.01;95% CI 0.7,1.4) but has a modest effect on pain at 2-7 days (OR 1.45;95% CI 1.1, 2.0). One needs to treat 21 children with antibiotics in order to prevent one child from experiencing pain after 2-7 days (Del Mar 1997). Untreated children are almost twice as likely as treated children to develop otitis media in the other ear (OR 1.75;95% CI 1.1,2.9), and 17 children would need to be treated early to prevent one child from developing infection in the other middle ear. Untreated children are no more likely as treated children to develop recurrent otitis media (OR 1.00;95% CI 0.8,1.3) (Glasziou 1999). Thus, treatment has some beneficial effects, but the NNT is relatively high. No treatment for some parents and children may be an appropriate option, particularly when pain or otalgia is minimal or when parents indicate a desire to restrict their children’s antibiotic use.

The other area of variation is in duration of treatment. A major concern currently in medicine is the development of antibiotic resistance, for which indiscriminate use of antibiotics is largely responsible (Dowell 1998). Available evidence indicates that 5 days of treatment are as effective as 10 days of treatment (particularly for children over the age of two), can lower the risk of antibiotic resistance, and can lower the cost of care (Kozyrskyj 1998).

- *Acute Sinusitis:* Acute sinusitis is a common respiratory infection in children and adolescents, with an estimated prevalence of 9.3% (7.7-10.9%) of children 1 to 5 years old seen, for any reason, in pediatrician's offices (Aitken 1998). There is substantial variation in the treatment of acute sinusitis with many practitioners treating acute infections for 21 days and often using more expensive, broader spectrum antibiotics. The focus of our intervention will be on the treatment of acute sinusitis with 10 days of low cost, relatively narrow spectrum antibiotics such as amoxicillin.

A recent meta-analysis (deFerranti 1998) examined 27 RCTs of antibiotic treatment of sinusitis. Risk of clinical failure among 1553 randomized patients was not meaningfully decreased with more expensive antibiotics compared to amoxicillin (RR 0.86, 95% CI .62, 1.19). The risk difference was 0.9 fewer failures per 100 patients, translating into a NNT of 111 patients with more expensive antibiotics to prevent one treatment failure compared to treatment with amoxicillin. In another meta-analysis, Low found that 10 days of antibiotics were effective in curing acute sinusitis and that longer courses were not needed (Low 1997).

- *Bronchiolitis:* Bronchiolitis is an acute, highly communicable lower respiratory tract infection, characterized by "cough, coryza, fever, expiratory wheezing, grunting, tachypnea, retractions and air trapping" (Welliver 1993). It has significant morbidity, accounting for 17% of all infant hospitalizations (9 admissions per 1000 child-years) in New York State (McConnochie 1995). Infants with bronchiolitis are wheezing for the first time, unlike asthmatics where wheezing is recurrent. It should be emphasized that there are differences in definition of bronchiolitis in different countries. In the United Kingdom and Australia, bronchiolitis refers to an illness starting as an upper respiratory infection followed by signs of acute respiratory distress and diffuse bilateral crepitations in addition to signs of bronchiolar obstruction such as air trapping and high pitched rhonchi (Disney 1960).

Bronchodilators are commonly used in the management of bronchiolitis. A Canadian study (Law 1993) found that 78% of those hospitalized with bronchiolitis received bronchodilators. A survey of pediatric allergists and pulmonologists in the United States (Newcomb 1989) found that 86% recommended a trial of bronchodilators for this condition. Similarly, in a survey of pediatric infectious disease specialists in Europe, the majority use bronchodilators for treatment of bronchiolitis (Kimpen 1997). However, bronchodilator efficacy for this illness is not universally accepted, and bronchodilators are seldom used to treat bronchiolitis in the United Kingdom (Goodman 1993).

Randomized clinical trials of bronchodilators in bronchiolitis, whether for ambulatory or hospitalized children, have yielded variable results.

A meta-analysis by Kellner et al indicates that bronchodilators can improve clinical wheezing (effect size = -0.2;95% CI -0.3,-0.1) but have no benefit on oxygen saturation (effect size = 0.02;95% CI -0.2,0.3) or risk of hospitalization (RR = 0.85;95% CI 0.5,1.5) (Kellner 1996;1999). Thus, we will propose that bronchodilators not be routinely prescribed in the outpatient setting.

- *Allergic Rhinitis:* Allergic rhinitis is a common condition, affecting approximately 12 million children and adolescents in the US (Malone 1997). The incidence of this problem has increased dramatically in recent years. Traditionally, treatment has been with oral anti-histamines. Newer classes of oral H1 receptor antagonists have been developed and are commonly used in the US such as loratadine and cetrizine. Another alternative treatment is intranasal steroids. A recent systematic review comparing these two treatments examined data on 2267 patients in 16 RCTs (Weiner 1998), and found that intra-nasal steroids were more effective at relieving nasal symptoms than were oral agents. This study found that intra-nasal steroids were more effective at relieving nasal symptoms including nasal blockage (standardized mean difference (SMD) = -0.63; 95% CI -0.7,-0.5), nasal discharge (SMD = -0.501;95% CI = -0.6,-0.4), sneezing (SMD = -0.49;95% CI -0.6,-0.4), nasal itch (SMD = -0.38;95% CI -0.5,-0.3), and postnasal drip (SMD = -0.24;95% CI -0.4,-0.1), than were oral agents. We propose to convert these standardized mean differences into more easily interpretable terms using pooled mean standard deviations from the studies referenced in the meta-analysis, and use the converted outcome data to encourage the use of intranasal steroids instead of oral decongestants for first line drug therapy of allergic rhinitis.

##### D.5.c Quality Assurance of evidence syntheses

Each of our interventions will be reviewed by an external panel of three physicians who are trained in evidence appraisal, in order to provide independent quality assurance. The physicians who have agreed to serve as external consultants are Tom Newman (University of California, San Francisco), John Frohna (University of Michigan), and Brett Robbins (University of Rochester, New York). Each of them have considerable experience in pediatric evidence based medicine. For each of the intervention screens that we have developed (or plan to develop), we will send the consultants the original article(s) upon which our evidence is based. We will ask them to familiarize themselves with the article, recommend other articles if necessary, and determine whether they think we have satisfactorily distilled the evidence into the bullet screen format. We will resolve differences by vote and/or negotiation.

**D.6 Outcome specification; patient population, sites and sources of data**

## D.6.a Pharmaceutical use

| Topic | Primary outcome | Patient population being studied | Data source |
| --- | --- | --- | --- |
| Acute otitis media | 1. % patients treated for ≤5 days compared to % treated for >5 days 2. % patients treated vs. % not treated | 1. All patients diagnosed with otitis media 2. All patients diagnosed with otitis media | 1. Automated capture of data elements from pharmacy and diagnostic fields, and clinic billing data   (2) Same |
| Acute sinusitis | 1. % of patients treated for ≤10 days compared to % treated for >10 days 2. % patients treated with Amoxicillin compared to % treated with other antibiotics | 1. All patients diagnosed with acute sinusitis 2. All patients diagnosed with acute sinusitis and treated with antibiotics | (1) & (2) Automated data capture as above |
| Bronchiolitis | (1) % patients diagnosed and not treated with b’dilators | (1) All patients diagnosed with bronchiolitis | (1) Automated data capture as above |
| Allergic rhinitis | (1) % patients diagnosed with allergic rhinitis and treated with intranasal steroids compared to % treated with oral agents | (1) All patients diagnosed with allergic rhinitis | (1) Automated data capture as above |

## D.7 Data analysis plan

## D.7.a Analysis of main effects

The effect of each intervention will be analyzed by comparing the percentage of patients receiving evidence-based care among providers randomized to the EBDSS with feedback to the percent among provider not receiving the intervention. The study design call for the analysis of patients clustered according to their provider. The analysis plan is therefore one which is appropriate for cluster randomization.

Logistic regression analysis will be used to test the change in percentage of patients receiving the specific type of care. Specifically, the proportion of patients receiving the outcome under study (e.g. for otitis media, the proportion receiving ≤5 days of therapy as opposed to >5 days of therapy) will be calculated per clinic site on a monthly basis, and the outcome rate among the patients whose providers are randomized to receive the intervention will be compared to the rate among the patients whose providers are randomized to not receive the intervention.

In the analysis, we will concomitantly adjust for other characteristics that might be different between the intervention groups, such as constant covariates (such as level of training, which might be difficult to adequately randomize beforehand using our stratified block randomization scheme), and varying covariates, such as average daily census. Data will be analyzed at the individual patient level in order to more thoroughly adjust for measurable individual patient characteristics, rather than would occur if we collapsed data by provider or by clinic. The final model will therefore provide an estimate of the effect of the intervention, summarized across providers and intervention sites, adjusted for relevant confounding patient and provider characteristics.

The analysis will need to account for the non-independence of clinic patients. To illustrate this, we believe it is plausible that patients at each of the clinic sites (such as the Family Medicine Clinic in the University of Washington Primary Care Center or the Skagit Pediatric clinic) are likely to be more similar to other patients attending that same clinic - in terms of health care behavior and willingness to change – than they are like patients attending clinics at the other sites. Any analysis of the rate of change of physician behavior will need to take into account this non-independence across sites. We will use the cluster routine available in STATA. The cluster variable will be specified by the individual provider, and will result in the variance estimates being adjusted accordingly, with the proper statistical test criteria being applied.

These analyses will be done individually for each of the rules representing a separate intervention. In addition, an overall measure of the decision support system will also be assessed by combining all interventions, and looking globally at the proportion of evidence-based decision making among providers randomized to receive the intervention, compared to the proportion among providers randomized to not receive the intervention.

## D.7.b Assessment of confounding and heterogeneity across sites

We will evaluate confounding by standard methodology. We will first enter the intervention effect into the model. Possible confounders (such provider level of training, season, or daily census) will be entered individually into the model, and those variables that substantively change the estimate of intervention effect will be retained in the final model. This process will be repeated until the addition of any further variables no longer substantively changes the intervention effect. We assume that it will be unnecessary to do considerable adjustment since this is a randomized clinical trial.

We will also assess the possibility that the decision support system might have a differentially strong effect at one site compared to another. We will assess this possibility of effect modification by looking for heterogeneity of the intervention odds ratios across the different intervention sites. If such heterogeneity exists, we will present the odds ratios separately by site, in order to demonstrate the difference in effects across sites.

## D.7.c Secondary analyses of (i) factorial design of otitis media rule and (ii) dissemination of intervention(s) among providers not receiving intervention

## In the analysis of the otitis media rule, we will be able to assess the added effect of the electronic feedback upon the rate of behavior change by analyzing the 2x2 factorial design described earlier in section D.3.c.2. To assess the added benefit of feedback to the EBDSS, we will compare the outcome among patients of providers receiving electronic feedback and the EBDSS to those receiving the EBDSS only. Similarly, to assess the added benefit of EBDSS to electronic feedback, we will compare the outcome among patients of providers receiving EBDSS and electronic feedback to those receiving electronic feedback only.

An additional aspect of our overall analytic plan is that we will be able to explicitly study the role of dissemination of information to providers not receiving the intervention. In this sub-analysis, we will assess the percent of patients receiving evidence-based care among providers randomized to *not* receive the EBDSS with feedback and compare this to the percent of patients receiving evidence-based care among these *very same* providers prior to the introduction of the EBDSS. If there is dissemination of the information even without direct use of the EBDSS and feedback, then we would expect that the proportion of patients receiving evidence-based care would increase even among physicians who are not randomized to the EBDSS with feedback. However, if dissemination does not occur, then we would expect that the proportion of patients receiving evidence-based care would remain more or less the same in the time period prior to and following introduction of the EBDSS.

# D.7.d Cost Analysis

The primary task of the cost analysis is to compare the costs of the program to the potential cost savings it generates to determine whether the intervention could in principle pay for itself. A secondary, but important, task of the cost analysis is to determine the net cost of the program per provider.

Cost savings are likely to accrue to the payer, the provider and the patient. The proportions of cost savings accruing to each of these groups will vary according to whether the health plan is a staff-model HMO, a group-model HMO with capitation, a traditional fee-for-service plan, or the patient’s deductible. To simplify the cost analysis and to promote its generalizability, the perspective of the cost analysis will be that of the contract between the provider and the payer. It will be assumed that this contract is subject to renegotiation if doing so could potentially reduce one party’s overall costs without raising the overall costs of the other. For example, in a capitated system, the reduction in number of visits for otitis media as patients learn about the modest therapeutic value of antibiotics would constitute a cost saving for the provider. On the other hand, in a staff-model HMO, this cost-savings would accrue to the payer. In either system, the contract between payer and provider could be written in such a way that the upfront and operating costs of the EBDSS would be split between them in proportion to their expected cost savings.

The advantages of this approach are that it generates implications for practice that are generalizable to all forms of provider-payer contracts, and that it identifies all potential net cost savings, including those which would require side payments to be incentive-compatible. This approach is standard in economics (Boardman 1996). The cost analysis will therefore determine whether the sum of the (discounted) cost-offsets to the payer and the provider exceed the (discounted) sum of the intervention costs to them.

# Costs

The costs of the intervention include (a) computers; (b) software development; (c) software maintenance; (d) periodic upgrades of evidence; (e) operating expenses (space, electricity); and perhaps (f) time (although the EBDSS is as likely to save time as to cost time). Estimates of some of these costs, such as operating expenses and periodic upgrades of evidence can be obtained in a straightforward manner from the associated costs in the study. For other costs, such as software development, large economies of scale imply that the costs of a real-world installation are likely to be very different than the costs incurred for this trial. Such costs will be estimated using per-user cost data on other, similar clinical software. Finally, the cost of the computer hardware will almost certainly undergo a secular declining trend, so that again the trial costs may be only approximately related to program costs in a real-world setting. For all such costs, the analysis will test the sensitivity of results to cost (as well as cost-offset) estimates using several standard techniques: one-way, multi-way, and probabilistic sensitivity analyses (Drummond 1997). Capital costs will be converted to annual equivalent expenditures using both 3% and 5% discount rates.

Many, but perhaps not all, of the hypothesized effects of the interventions on the individual outcomes described in section D.6 will result in cost savings. For example, the EBDSS may discourage providers from prescribing antibiotics in certain cases of acute otitis media, resulting in a cost savings. On the other hand, cautious providers who reduce antibiotic use might increase the scheduling of follow-up visits to be certain the condition has resolved itself on its own. The cost analysis will determine whether EBDSS results in a shift to lower-cost care overall.

# Cost Offsets

The cost offsets expected as a result of the intervention arise from changing physician and patient behaviour from more costly to less costly (but equally good or better) treatment options. For example, the EBDSS is hypothesized to change prescribing behavior for Otitis Media from longer treatment durations with antibiotics to shorter treatment durations, and from antibiotics to no treatment. In both cases, immediate cost savings will be realized. In addition, future cost savings might be realized because fewer patients can be expected to return for treatment for symptoms associated with the side effects of antibiotic use.

A schema for evaluating this hypothesis is presented in Figure 1, which depicts a decision tree for the diagnosis and treatment of the topics covered in this proposed intervention. The branching of the decision tree represents in most cases decisions made by the provider in conjunction with the patient’s parents, and in some cases morbidity outcomes. For each branch we present the probability of the branch conditional on having reached the previous node, using the baseline data. For example, among patients with acute OM, 75% were treated with antibiotics, while 25% received no treatment. By multiplying out these conditional probabilities, it is possible to obtain the probability of each individual branch, presented in a column at the right margin of the Figure.

Cost data will be recovered from several sources. Charges for visits are available from charge schedules for each of the study sites. Inferences about converting charges to costs will be drawn by comparing charge schedules at the clinics to allowable fee schedules and capitation rates of the Washington State Department of Social and Health Services and Medicare.

The cost of treatment along each branch will be assumed to be the same in the intervention and control groups. What will change is the probabilities of each branch. Multiplying the probabilities of the branches by the costs of the branches and summing the products and dividing by the number of visits yields the expected costs of a visit. The difference in costs between the intervention and control groups can then be assessed to determine annual cost-savings. Thus, the main cost savings to be realized in the intervention will be the shift in branching probabilities resulting from the intervention.

During the grant period, the researchers will quantify potential additional cost offsets using the secondary literature. For example, it may not be possible within the time-frame of the trial to ascertain longer-term effects of alternative treatment options, such as adverse effects from antibiotics or bronchodilators. However, the cost implications of such side effects can be large, and important cost savings could be realized if the intervention occasions a shift in treatment behavior away from prescriptions of drugs with significant adverse effects toward safer drugs.

Other possible long-term cost offsets include the potential for fewer clinical visits as parents learn to identify conditions in which no treatment is the best option, the benefits of more legible prescriptions, and the potential reduction of risk of adverse drug interactions (Winslow 1997; Davis 1994;Ishizuka 1986).

As can be seen from Figure I, the intervention covers conditions seen in 12.1% of pediatric visits in the baseline data. Thus, the intervention in this trial has a broad scope relative to its eventual full implementation in a real-world application. Of course, broadening the scope to include other conditions would increase the benefits (or cost-offsets) of the intervention, while entailing only modest incremental costs. These issues will be explored in greater detail during the grant period.

Figure I: Decision Tree for the Diagnosis and Treatment

# D.8 Power and Sample Size Calculations

The power analysis presented here focuses on the outcome of prescription of antibiotics for otitis media. Such visits compose about 8.4% of the caseload at the Pediatric Clinic at the University of Washington Primary Care Center. The power of the trial to detect changes in provider behavior over the first six months of the study is assessed. The power to detect a given treatment effect over the full two years of the trial period will be similar for allergic rhinitis (2.5% of visits) and acute sinusitis (1.6% of visits) outcomes, and somewhat worse at detecting changes in bronchiolitis outcomes (0.6%). However, for all conditions, power given these numbers of observations is much more sensitive to the standard deviation of the outcome across providers than to the numbers of providers or visits.

Currently, approximately 75% of cases of otitis media at the Pediatric Clinic at the University of Washington Primary Care Center were treated with antibiotics. For purposes of this power calculation, we have conservatively estimated that the treatment effect will be to lower this proportion to approximately 60%, a difference of 15 percentage points.

The intervention studied here is applied at the level of the provider, but outcomes are measured at the level of the visit. This distinction implies that the statistical analysis—starting with the power calculations—will have to consider clustering effects. In this design, each provider is a cluster, and the providers’ visits are the observed outcomes. Power was adjusted for clustering effects using the method of Hayes and Bennett (1999).

The estimates of power presented here assume that 42 providers are randomized to each of the two study arms, and that each provider sees an average of 30 cases of otitis media over the first six months of the study period.

Figure 2: Power to detect changes in physician prescribing behavior for Otitis Media

Because of the possible importance of cluster effects, the power of the study depends not only on the average number of visits per provider, but also on the distribution of those visits across providers. (Specifically, a more equal distribution of visits across providers yields greater power *ceteris paribus* than a distribution of visits heavily weighted to a few providers.) Existing distributions of visits at the three study site clinics were employed to make assumptions about the likely future distributions of such visits there. Every effort was made to be conservative in this extrapolation.

The figure below presents the results of a sensitivity study of the power of this trial to detect a variety of treatment effects under a variety of assumptions about the standard deviation of the mean outcome across providers. Treatment effects were varied from a 5 percentage-point shift in mean outcomes to a 25 percentage-point shift. Standard deviations were varied from 0.05 to 0.25. The best *a priori* guess for the actual standard deviation in the trial is 0.10 - 0.15, and the best *a priori* guess for the treatment effects will be between 10 and 20 percentage points, depending on the outcome.

As can be seen, there is a wide range of power associated with different assumptions about the standard deviation of the mean outcome variable across providers (σ_c_). The study will be underpowered to detect very small treatment effects of less than 5%, or larger effects if the standard deviation of outcomes across providers is very large. In the domain of the best *a priori* guesses for these variables, power ranges from 40% to 99.9%. A treatment effect of 0.15 can be detected with 90% power if the standard deviation of the outcome across providers is 0.15.We plan to conduct interim analyses at 6 months, 1 year, and 18 months to assess the efficacy of this trial and to consider stopping it for various interventions depending on the magnitude and significance of the effect. We anticipate having more than adequate power at the first analysis to make a decision regarding the otitis intervention although we are uncertain about the others. Although the risks of the intervention are minimal, because there may be repeated analyses for some of the interventions, we will follow the early stoppage rules as described by O’Brien and Fleming.

## D.9 Timetable

| Study Timeline | | | | | | | | | | | | |
| --- | --- | --- | --- | --- | --- | --- | --- | --- | --- | --- | --- | --- |
| May-00 | Aug-99 | Nov-00 | Feb-00 | May-01 | Aug-00 | Nov-01 | Feb-01 | May-02 | Aug-01 | Nov-02 | Feb-02 | May-03 |
|  |  |  |  |  |  |  |  |  |  |  |  |  |
| Personnel Hiring | | |  |  |  |  |  |  |  |  |  |  |
|  |  |  |  |  |  |  |  |  |  |  |  |  |
|  | Computer Installation | | |  |  |  |  |  |  |  |  |  |
|  |  |  |  |  |  |  |  |  |  |  |  |  |
|  |  | Baseline Data Collection | | | | | |  |  |  |  |  |
|  |  |  |  |  |  |  |  |  |  |  |  |  |
|  |  |  |  | EBDSS Pharmaceutical Intervention & Data Collection | | | | | | | |  |
|  |  |  |  |  |  |  |  |  |  |  |  |  |
|  |  |  |  |  |  |  |  | Trial Dataset Preparation | | | |  |
|  |  |  |  |  |  |  |  |  |  |  |  |  |
|  |  |  |  |  |  |  |  |  | Dataset Preparation & Analysis | | | |
|  |  |  |  |  |  |  |  |  |  |  |  |  |

## D.10 Dissemination

In addition to the usual vehicles employed for dissemination of project results-e.g. peer-reviewed publications and conference presentations, we expect that the results of this study will be disseminated through the efforts of the AHCPR. Moreover, because our goal is to change physician practice and improve evidence based child health, we will also ensure that local dissemination efforts are maximized. We plan to present our findings to key decision makers at various health care delivery systems in the area. During these presentations, we will use each participating clinic as a leverage point to gain cooperation of other affiliated sites. For example, success at the semi rural clinic may convince one of the Seattle urban clinics affiliated with a large health care system in the Seattle area (Virginia Mason) to use our system (they have already expressed preliminary interest in possibly using our system). We will make a series of presentations to participating and non-participating practices and delivery systems within the greater Seattle area to maximize the local effects of our efforts. In addition, at an evidence-based pediatric conference that we are in the process of planning for next winter, we will showcase the EBDSS so as to demonstrate to the visiting clinicians, academicians, and managed care executives how the system operates and what it has to offer.

## D.11 Limitations and considerations

It would be desirable to perform all the interventions within this study in the context of a randomized clinical trial, where patients were the unit of randomization. However, there are at least two important reasons not to apply the intervention directly to the patient (i.e. randomize patients within providers). First, pragmatically this would entail turning the EBDSS on for some patients and off for others, and then assessing treatment difference between the two. It is unlikely that we would be able to employ such rapid randomization techniques that would requiring the EBDSS to be activated for some patients and deactivated for others. Second, it is also likely that a clinician will retain at least some knowledge from past use of the EBDSS with previous patients, and by applying this to all or most subsequent patients, this contamination would again lead to an underestimate of the effect of the EBDSS.

It is possible, although unlikely, that the analysis of the dissemination to providers not receiving the intervention (outlined in D.7.c) might be compromised as a result of events outside the study. For example, should the American Academy of Pediatrics release a position statement on acute sinusitis concomitant with our release of this particular intervention, we could see a change in practice among the providers not receiving the intervention, and this change would not necessarily be due to dissemination of our intervention. Therefore, we plan to implement our modules sequentially (rather than implement all at once); it is highly unlikely that external events would coincide with each module implementation. Nevertheless, the principal investigators will remain aware of any external events that might influence our study (such as media campaigns to reduce antibiotic use, etc), and we will assess the results of our study accordingly.

## D.12 Gender and Minority

All sites see patients of all sex, race, and ethnic groups. Medicaid patients are seen at each site. The gender, racial and ethnic make-up of the populations seen at each site is roughly proportional to their representation in Washington State. Members of each gender and racial/ethnic group will be included in this study without any barriers whatsoever. In King County, the racial/ethnic makeup of the population under 17 years of age is approximately 78% white, 8% black, 12.5% Asian/Pacific Islander, and 1.5% Native American. In Washington State, the make-up is 86% Caucasian, 4.5% Black, 7% Asian/Pacific Islander, and 2.5% Native American. With respect to the practitioners at the Pediatric Clinic at the University of Washington Primary Care Center, 1 is African-American, 4 are Asian and 35 are Caucasian. Eighteen of the clinicians are female. We believe that the breakdowns at the other sites are similar. We have made no exclusions based on race/ethnicity at any of our sites.

## E. Human subjects considerations

This project differs slightly from present practice in the sense that prescription writing will be done on-line rather than manually, and evidence will be provided to providers at the time that these tasks are performed. Because we view this as a quality improvement project, individual patients will not be approached for consent. All providers at each clinic site have agreed to participate. There are no serious risks to providers or patients as a result of this study. Providers may or may not choose to follow the recommendations provided to them. In general, on line prescribing, with clearly printed prescriptions and legible names has been shown to decrease the risk of adverse drug reactions, particularly with respect to dosing errors. The data to be collected will be stripped of identifiers after production of the analysis dataset. The Children’s Hospital and Regional Medical Center Institutional review board has reviewed and approved this study (Reference IRB Number: 155-99-06 IRB Approval Period: 6/22/99 - 6/21/2000).

## G. Literature Cited

Aitken M, Taylor JA. Prevalence of clinical sinusitis in young children followed up by primary care pediatricians. Arch Pediatr Adolesc Med. 1998; 152: 244-8.

Alho OP, Koivu M, Sorri M, Rantakallio P. The occurrence of acute otitis media in infants. A life-table analysis. Int J Pediatr Otorhinolaryngol. 1991; 21: 7-14.

Antman EM, Lau J, Kupelnick B, Mosteller F, Chalmers TC. A comparison of results of meta-analyses of randomized control trials and recommendations of clinical experts. Treatments for myocardial infarction. *JAMA*. 1992;268:240-8.

Atiyeh BA, Dabbagh SS, Gruskin AB. Evaluation of renal function during childhood. Pediatr Rev 1996 May;17(5):175-80

Avorn J, Soumerai SB. Improving drug-therapy decisions through

educational outreach. A randomized controlled trial of academically based

"detailing". N Engl J Med 1983; 308(24):1457-63

Bates DW, Leape LL, Cullen DJ, et al. Effect of computerized physician order entry and a team intervention on prevention of serious medication errors. *JAMA*. 1998;280:1311-6.

Bero LA, Grilli R, Grimshaw JM, et al. Closing the gap between research

and practice: an overview of systematic reviews of interventions to promote

the implementation of research findings. BMJ 1998;317:465-8.

Boardman AE, Greenberg DH, Vining AR, Weimer DL. Cost-Benefit Analysis: Concepts and Practice. Upper Saddle River: Prentice Hall. 1996.

Chueh H, Barnett GO. "Just-in-time" clinical information*. Acad M*ed. 1997;72:512-7.

Christakis DA, Rivara FP Pediatricians' awareness of and attitudes about four clinical practice guidelines. Pediatrics 1998 May;101(5):825-30.

Davis NM. Confusion over illegible orders. Am J Nurs. 1994:94(1):9

deFerranti SD, Ioannidis JPA, lau J, Anninger WV, Barza M. Are amoxicillin and folate inhibitors as effective as other antibiotics for acute sinusitis? A meta analysis. BMJ 1998; 317:632-7.

Del Mar C, Glasziou P, Hayem M. Are antibiotics indicated as initial treatment for children with acute otitis media? A meta-analysis. BMJ. 1997; 314: 1526-9.

Disney ME, Sandiford BR, Cragg J, Wolff J. Epidemic bronchiolitis in infants. BMJ. 1960; 1: 1407-1411

Dowell SF, Marcy SM, et al. Principles of judicious use of antimicrobial agents for pediatric upper respiratory tract infections. Pediatrics. 1998; 101(supp 1):163-5.

Drummond MF, O’Brien B, Stoddart GL, Torrance GW. Methods for the Economic Evaluation of Health Care Programs. Oxford: Oxford University Press. 1997.

Evans RS, Pestotnik SL, Classen DC, et al. A computer-assisted management program for antibiotics and other antiinfective agents. *N Engl J Med*. 1998;338:232-8.

Fonseca MT, Camargos PA, Ferreira CS, Filogonio CB, Pitchon R, Viotti VN, Maciel PE, Castro TA Interobserver agreement in assessing plain radiographs of maxillary sinus. **Int J Pediatr Otorhinolaryngol** 1998 Sep 15;45(1):41-6

Forrest CB, Simpson L, Clancy C. Child Health Service Research; Challenges and Opportunities. JAMA 1997;277:1787-1793.

Gabay C, Kushner I. Acute-phase proteins and other systemic responses to inflammation. NEJM 1999; 340:448-454.

Gerber MA, Tanz RR, Kabat W et al. Optical immunoassay test for GABHS pharyngitis: an office based, multicenter investigation. JAMA 1997; 227:899-903.

Glasziou PP, Hayem M, Del Mar CB. Antibiotic versus placebo for acute otitis media in children (Cochrane Review). In: The Cochrane Library, Issue 1, 1999. Oxford: Update Software.

Goodman BT, Chambers TL. Bronchodilators for bronchiolitis? Lancet. 1993; 341:1380.

Greco PJ, Eisenberg JM. Changing physicians' practices. *N Engl J Med*. 1993;329:1271-3.

Grol R, Dalhuijsen J, Thomas S, Veld C, Rutten G, Mokkink H. Attributes of clinical guidelines that influence use of guidelines in general practice: observational study. BMJ 1998 Sep 26;317(7162):858-61

Hayes RJ and Bennett S. Simple sample size calculation for cluster-randomized trials. Int J Epidem 1999; 28:319-326.

Hayes RJ and Bennett S. Simple sample size calculation for cluster-randomized trials. Int J Epidem 1999; 28:319-326.

Hunt DL, Haynes RB, Hanna SE, Smith K. Effects of computer-based

clinical decision support systems on physician performance and patient

outcomes: A systematic review. JAMA 1998;280(15): 1339-46.

Ishizuka H, Hara S, Ishikura C. Computerized prescription checking system. Int J Biomed Comput. 1986:19(3-4):195-200.

Johnson KB, Feldman MJ. Medical informatics and pediatrics. Decision-support systems. *Arch Pediatr Adolesc Med*. 1995;149:1371-80.

Johnston ME, Langton KB, Haynes RB, Mathieu A. Effects of computer-based clinical decision support systems on clinician performance and patient

outcome: A critical appraisal of research. Ann Intern Med 1994; 120(2):

135-42.

Kellner JD, Ohlsson A, Gadomski AM, Wang EEL. Bronchodilator Therapy in Bronchiolitis (Cochrane Review). In: The Cochrane Library, Issue 1, 1999. Oxford: Update Software.

Kellner JD, Ohlsson A, Gadomski AM, Wang EEL. Efficacy of Bronchodilator Therapy in Bronchiolitis: A Meta-analysis. Arch Pediatr Adolesc Med 1996; 150: 1166-1172.

Kimpen JLL, Schaad UB. Treatment of respiratory syncytial virus bronchiolitis: 1995 poll of members of European Society for Pediatric Infectious Diseases. Pediatr Infect Dis J 1997; 16: 479-481.

Klein JO. Epidemiology of otitis media. Pediatr Infect Dis J. 1989;8(Suppl):S9.

Kozyrskyj AL, Hildes-Ripstein GE, et al. Treatment of acute otitis media with a shortened course of antibiotics: A meta-analysis. JAMA. 1998; 279:1736-42.

Laine K, Maatta T, Varonen H, Makela M. Diagnosing acute maxillary sinusitis in primary care: a comparison of ultrasound, clinical examination and radiography. **Rhinology** 1998 Mar;36(1):2-6

Law BJ, De Carvalho V, et al. Respiratory syncytial virus infections in hospitalized Canadian children: regional differences in patient populations and management practices. Pediatr Infect Dis J 1993; 12: 659-63.

Lohr JA, et al. Making a presumptive diagnosis of a UTI by using a urinalysis performed in an on-site lab. J Pediatrics 1993; 122: 22-25.

Low DE. 10 days of amoxicillin is effective for acute sinusitis. Can Med Assoc J 1997; 156:1S-14S

Malone DC, Lawson KA, Smith DH, et al. A cost of illness study of allergic rhinitis in the United States. J Allergy Clin Immunol. 1997; 99: 22-7.

McCaig LF, Hughes JM. Trends in antimicrobial prescribing among office-based physicians in the United States. JAMA 1995; 273: 214-9.

McConnochie KM, Roghmann KJ, Liptak GS. Hospitalization for lower respiratory tract illness in infants: variation in rates among counties in New York State and areas within Monroe County. J Pediatr 1995; 126: 220-9.

Newcomb RW. Use of adrenergic bronchodilators by pediatric allergists and pulmonologists. Am J Dis Child 1989; 143: 481-5.

Nyquist AC, Gonzales R, Steiner JF, Sande MA. Antibiotic prescribing for children with colds, upper respiratory tract infections, and bronchitis. *JAMA*. 1998;279:875-7.

O’Brien PC, Fleming TR. A multiple testing procedure for clinical trials. Biometrics 35:549-556

Oxman AD, Thomson MA, Davis DA, Haynes RB. No magic bullets: a systematic review of 102 trials of interventions to improve professional practice. *CMAJ*. 1995;153:1423-31.

Pennie RA. Prospective study of antibiotic prescribing for children. *Can Fam Physician*. 1998;44:1850-6.

Pestotnik SL, Classen DC, Evans RS, Burke JP. Implementing antibiotic practice guidelines through computer-assisted decision support: clinical and financial outcomes. *Ann Intern Med*. 1996;124:884-90.

Raschke RA, Gollihare B, Wunderlich TA, et al. A computer alert system to prevent injury from adverse drug events: development and evaluation in a community teaching hospital. *JAMA*. 1998;280:1317-20.

Shortliffe E. Testing reality: the introduction of decision-support technolgies for physicians. *Methods Inf Med*. 1989;28:1-5.

Solomon DH, Hashimoto H, Daltroy L, Liang MH. Techniques to Improve Physicians’ Use of Diagnostic Tests. JAMA 1998;280:2020-2027.

Stool SE. Evolution of pediatric otolaryngology. Pediatr Clin North Am. 1989; 36: 1363-9.

Tarczy-Hornoch, P., Kwan-Gett, T.S., Fouche, L., Hoath, J., Fuller,

#### S., Ibrahim, K., Ketchell, D.S., LoGerfo, J.P., Goldberg, H.I. "Meeting

#### Clinician Information Needs by Integrating Access to the Medical Record

and Knowledge Resources via the Web". Jour Amer Med Inform Assoc, Fall

#### Symposium Suppl, 809-813,1997

van Walraven C, Goel V, Chan B. Effect of population based interventions on lab utilization. JAMA 1998; 280:2028-2033.

Wald ER, Reilly JS, Casselbrant MC, Chiponis DM. Treatment of acute sinusitis in children with augmentin vs cefaclor. Postgrad Med 1984;Sept-Oct: 133-6.

Webb KH. Does culture confirmation of high-sensitivity Rapid Streptococcal Tests make sense? A medical decision analysis. Pediatrics 1998; 101:2/e2

Weinberg AG, Gan VN. Urine screen for bacteriuria in symptomatic pediatric outpatients. Pediatr Infect Dis 1991; 10: 651-4.

Weiner JM, Abramson MJ, Puy RM. Intranasal corticosteroids versus oral H_1_ receptor antagonists in allergic rhinitis: systematic review of randomised controlled trials. BMJ 1998;317:1624-1629

Welliver JR, et al. Bronchiolitis. Pediatr Rev. 1993;14:134-9.

Winslow EH, Nestor VA, Davidoff SK, Thompson PG, Borum JC. Legibility and completeness of physicians’ handwritten medication orders. Heart Lung 1997:26(2):158-64.

H. Contractual Agreements

This proposed study is a collaborative project between researchers at the University of Washington and the above named intervention sites. A subcontract agreement will be established with the CHRMC. The research team at the University of Washington (led by Dr. Davis) will be responsible for coordinating efforts with the intervention sites, developing and distributing the various components of the EBDSS and electronic feedback, for collecting and analyzing study data, and handling budget and other administrative aspects of managing the grant.

1. Consultants

Each of our interventions will be reviewed by an external panel of three physicians who are trained in evidence appraisal, in order to provide independent quality assurance. The physicians who have agreed to serve as external consultants are Tom Newman (University of California, San Francisco), John Frohna (University of Michigan), and Brett Robbins (University of Rochester, New York). Each of them have considerable experience in pediatric evidence based medicine. For each of the intervention screens that we have developed (or plan to develop), we will send the consultants the original article(s) upon which our evidence is based. We will ask them to familiarize themselves with the article, recommend other articles if necessary, and determine whether they think we have satisfactorily distilled the evidence into the bullet screen format. We will resolve differences by vote and/or negotiation.
