# Supplementary figures and images for: A Cluster Randomized Clinical Trial to Improve Prescribing Patterns in Ambulatory Pediatrics
Source: PLoS Clin Trials. 2007 May 18;2(5):e25. doi: 10.1371/journal.pctr.0020025 (PMC1876598; doi:10.1371/journal.pctr.0020025)

## Slide 1
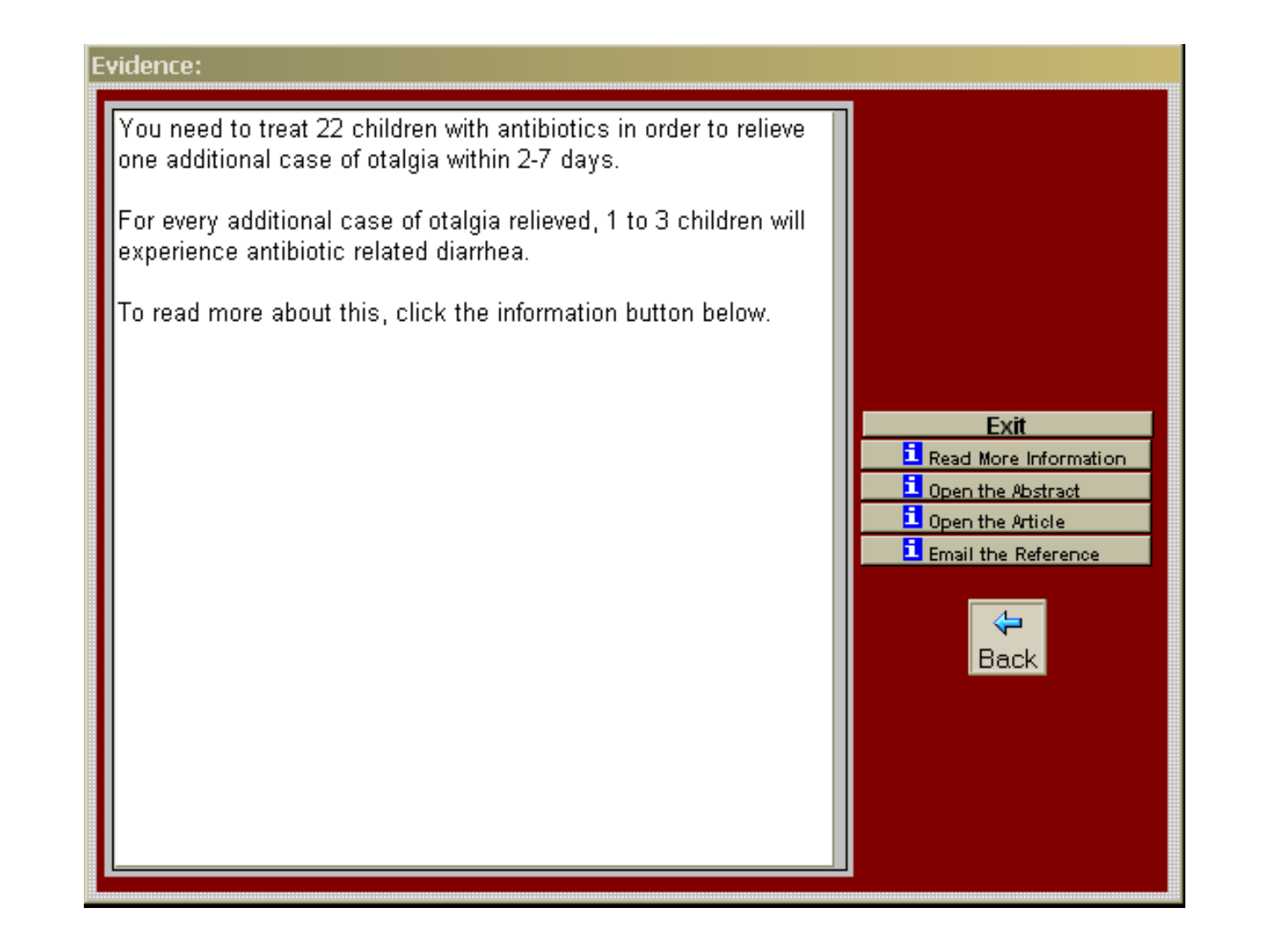

Supplement: Figure S1 — (38 KB PPT) [file pctr.0020025.sg001.ppt]
